# Mucosal vaccination with long-form TSLP induces migratory cDC1-mediated adaptive immunity against SARS-CoV-2 infection

**DOI:** 10.1128/jvi.01231-25

**Published:** 2025-08-19

**Authors:** Jing Hu, Housheng Zheng, Wei Ran, Xuefei Wang, Chenghui Liao, Jian Zhou, Liang Ye

**Affiliations:** 1 Department of Immunology, International Cancer Center, Shenzhen University Medical School, Shenzhen University 47890, Shenzhen, China; 2 The First Affiliated Hospital of Guangzhou Medical University, Guangzhou Medical University 26468, Guangzhou, China; St Jude Children's Research Hospital, Memphis, Tennessee, USA

**Keywords:** vaccination, SARS-CoV-2, thymic stromal lymphopoietin, dendritic cell, germinal center

## Abstract

A combination of vaccination strategies will potentially be required for effective control of the virus pandemic. We report that mice intranasally immunized with commercial severe acute respiratory syndrome coronavirus 2 (SARS-CoV-2) subunit vaccines enriched with the human thymic stromal lymphopoietin (TSLP) variant, long-form TSLP (lfTSLP), but not the short-isoform TSLP (sfTSLP), induced robust antigen-specific systemic and mucosal antibody production. The adjuvant-enhancing activity of lfTSLP in mice requires functional TSLP receptor signals in migratory type 1 conventional dendritic cells (cDC1s). Furthermore, lfTSLP acts on migratory cDC1s to enhance T follicular helper (Tfh) cell and germinal center (GC) B cell responses. Intranasal vaccination with lfTSLP elicits long-lasting immunogenicity and protection against the challenge of wild-type SARS-CoV-2 and the B.1.617.2 variant in mice. Our study provides insights into the adjuvant role of lfTSLP, which is critical in enhancing migratory cDC1-mediated GC responses to improve vaccine efficacy.

**IMPORTANCE:**

Adjuvants are indispensable components of subunit vaccines, and the development of adjuvants capable of inducing powerful systemic and mucosal immune responses is critical for enhancing the efficacy of viral vaccines. This study reveals that the human long-form thymic stromal lymphopoietin (lfTSLP) induces antigen-specific systemic IgG and mucosal IgA antibody production with sustained immunogenicity. Mechanistically, lfTSLP enhances germinal center reactions by preferentially activating migratory type 1 conventional dendritic cells (cDC1s). These findings uncover a previously unrecognized mechanism underlying the adjuvant activity of lfTSLP, which enhances vaccine-induced adaptive immunity and confers protection against SARS-CoV-2 infection. These findings indicate that the application of lfTSLP as an adjuvant should be encouraged in the rational design and development of viral vaccines.

## INTRODUCTION

Vaccination with subunit vaccines has provided considerable protection against severe diseases, including influenza, hepatitis B, and coronavirus disease 2019 (COVID-19), yet subunit vaccine-induced immune responses and efficacy are frequently insufficient, particularly in young and elderly populations (1, 2). The adjuvant is a crucial component of subunit vaccines that augments and orchestrates immune responses for protection against viruses (3–5). Currently, the development of adjuvants that enable robust systemic and mucosal adaptive immune responses is expected to improve the effectiveness of viral vaccines (6, 7).

Thymic stromal lymphopoietin (TSLP) is a four-helical type I cytokine produced by multiple cell types, including epithelial cells and dendritic cells (DCs) (8, 9). TSLP exerts its biological functions by binding to a high-affinity heteromeric surface receptor consisting of the TSLP receptor (TSLPR) and interferon-7 receptor α chain (IL-7Rα) in both humans and mice (8, 10, 11). As TSLP binds to its receptor, it stimulates Janus kinase 1 (JAK1) and JAK2, which then activate signal transducer and activator of transcription 5 (STAT5), promoting the transcription of target genes (8, 10, 11). TSLPR exists on diverse immune cells, including dendritic cells (DCs), T cells, and B cells (8, 9). TSLP interacts with TSLPR to modulate immune responses to respiratory viral infections, such as influenza virus and respiratory syncytial virus (RSV) (4, 12, 13). We and other research groups reported that mouse TSLP (mTSLP) was identified as a potential novel mucosal adjuvant (4, 14–17). mTSLP enhances influenza and severe acute respiratory syndrome coronavirus 2 (SARS-CoV-2) vaccine-mediated adaptive mucosal immunity (15, 18). Administered with human immunodeficiency virus (HIV) envelope proteins supplemented with mTSLP, it produced robust mucosal and systemic antibodies in mice (16). Furthermore, our previous findings demonstrated that endogenous TSLP signaling is required for the mucosal adjuvant activity of interferon-λ (IFN-λ) after intranasal immunization (4, 15, 19, 20).

Recent studies identified two variants of TSLP in humans: the long-form TSLP (lfTSLP) and the short-form TSLP (sfTSLP), whereas mice only have full-length TSLP (21). lfTSLP contains 159 amino acid sequences, while sfTSLP contains 63 amino acids that overlap with the C terminus of lfTSLP (21). lfTSLP expression was identified in inflammatory conditions through induction by TLR ligands, whereas sfTSLP is mainly continuously expressed in normal tissues (21, 22). Most studies do not distinguish between lfTSLP and sfTSLP, simply referring to both as TSLP. Although mice do not express sfTSLP, numerous investigations show that sfTSLP plays a crucial role in various mouse models, including reducing airway inflammation, inhibiting autophagy and remodeling, improving experimental colitis, and preventing endotoxin shock (23–27). Furthermore, few current studies indicate that lfTSLP and sfTSLP may have opposite biological functions, with lfTSLP exerting pro-inflammatory effects and sfTSLP exerting anti-inflammatory effects (22–24). However, whether lfTSLP and sfTSLP can be used as adjuvants to improve vaccine effectiveness—and what their mechanisms of action are—remains poorly understood.

We researched the role of lfTSLP and sfTSLP in the adaptive immune responses following intranasal immunization with commercial SARS-CoV-2 subunit vaccines and revealed that lfTSLP, but not sfTSLP, is essential for enhancing vaccine-specific IgG1 and IgA production and promoting long-term immunogenicity. lfTSLP primarily acts on migratory type 1 conventional dendritic cell (cDC1) subtypes to augment Tfh cells and germinal center (GC) B cell responses. This previously unexplored adjuvant activity of lfTSLP is responsible for improving vaccine-induced adaptive immunity and protecting against SARS-CoV-2 infection.

## RESULTS

### lfTSLP induces robust antibody response upon intranasal vaccination in a TSLPR-dependent manner

As TSLP performs its biological roles by binding to the TSLP receptor (TSLPR), our initial investigation aimed to determine whether human TSLP isoforms can act on mouse cells via mouse TSLPR (mTSLPR) signaling. We observed that lfTSLP- and sfTSLP-treated mouse bone marrow-derived dendritic cells (BMDCs) exhibited increased phosphorylation of STAT5 (Fig. S1A), but it was completely abrogated in TSLPR-deficient BMDCs (Fig. S1B), indicating that lfTSLP and sfTSLP might have biological roles in mice.

**Fig 1:**
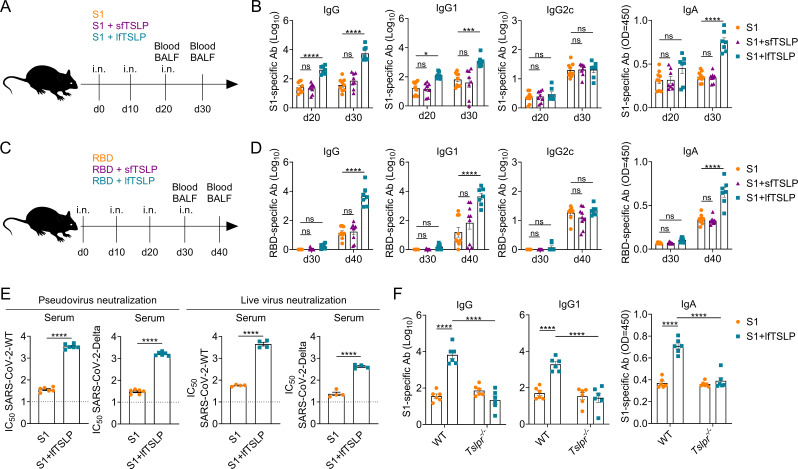
lfTSLP, but not sfTSLP, induces TSLPR-dependent robust antibody responses after intranasal administration. (**A and B**) WT mice were intranasally immunized with S1 in the presence or absence of sfTSLP or lfTSLP, and titers of serum S1-specific IgG subclasses on day 10, after one and two booster immunizations, were determined by ELISA. BALF IgA levels were measured 10 days after the first and second booster immunizations. *n* = 7–9 mice per group. (**C and D**) WT mice were immunized by intranasal application of RBD in the presence or absence of sfTSLP or lfTSLP. Ten days after the second and third boost immunizations, serum and BALF were analyzed for RBD-specific IgG subclasses and IgA by ELISA. *n* = 7–9 mice per group. (**E**) WT mice were intranasally immunized with S1 in the presence or absence of lfTSLP, and neutralization of serum antibodies to the SARS-CoV-2 (WT) and B.1.617.2 (Delta) pseudotyped viruses and authentic viruses was assessed by ELISA on day 10 after two booster immunizations. *n* = 4–6 mice per group. (**F**) WT (*n* = 6) and *Tslpr^–/–^* mice (*n* = 6) were immunized intranasally with S1 in the presence or absence of lfTSLP. Serum and BALF on day 10 after two booster immunizations were analyzed for S1-specific IgG, IgG1, and IgA by ELISA. ns, no significant difference. Results are representative of three independent experiments and shown as mean ± SEM. *P < 0.05, ***P < 0.001, ****P < 0.0001, by two-way ANOVA with Tukey’s multiple-comparison test (**B–D, F**) or unpaired two-tailed Student’s *t*-test (**E**). Each symbol represents an individual animal.

To investigate the role of lfTSLP and sfTSLP in vaccine-induced adaptive immunity, we intranasally immunized mice with 3 µg of commercial SARS-CoV-2 (wild-type strain) spike protein S1 subunit (S1) supplemented with 4 µg of lfTSLP or 4 µg of sfTSLP on days 0, 10, and 20, adhering to the previously reported immunization schedule (4, 15, 20). Ten days after the first and second booster immunizations, S1- or SARS-CoV-2 receptor-binding domain (RBD)-specific IgG subclasses in serum and S1- or RBD-specific IgA in bronchoalveolar lavage fluid (BALF) were evaluated by ELISA (Fig. 1A; Fig. S2A). Intranasal administration of S1 alone only resulted in low S1- or RBD-specific serum IgG titers, whereas applying lfTSLP to the vaccination resulted in a significant increase in S1- or RBD-specific serum IgG levels (Fig. 1B; Fig. S2B). When S1 was formulated with lfTSLP, serum S1- or RBD-specific IgG1 levels, but not IgG2c, were remarkably increased compared to control mice (Fig. 1B; Fig. S2B). Moreover, lfTSLP dramatically promoted BALF S1- or RBD-specific IgA levels 10 days after the second booster vaccinations (Fig. 1B; Fig. S2B). However, no enhancing effect was observed when S1 was formulated with sfTSLP after intranasal immunization (Fig. 1B; Fig. S2B). In line with these results, lfTSLP, but not sfTSLP, improves serum IgG and IgG1 and BALF IgA responses when the 5 µg RBD is administered intranasally 10 days after the third booster immunization (Fig. 1C and D), suggesting that intranasal vaccination with the lfTSLP rather than the sfTSLP-adjuvanted vaccine can induce robust antigen-specific IgG1 and IgA production. We then evaluate the functional efficiency of lfTSLP-induced neutralizing antibody levels on day 10 after second booster immunizations. We found that the serum neutralizing antibody elicited by the lfTSLP-complemented vaccine was effective against SARS-CoV-2 WT and Delta pseudoviruses and authentic viruses (Fig. 1E). However, the serum neutralizing antibodies against these two strains were rather weak in mice administered S1 alone (Fig. 1E).

To further explore whether lfTSLP-induced humoral immunity is dependent on TSLPR, we intranasally immunized WT and *Tslpr*^–/–^ mice with S1 with or without lfTSLP. In *Tslpr*^–/–^ mice, lfTSLP failed to improve S1-specific IgG and IgG1 levels in serum (Fig. 1F). Consistent with these results, poor IgA responses were elicited by S1 formulated with lfTSLP in *Tslpr*^–/–^ mice after intranasal immunization (Fig. 1F), indicating that the functional TSLP receptors are specifically required for the enhancing effect of lfTSLP-adjuvanted mucosal vaccines.

### lfTSLP amplifies GC responses after intranasal immunization via TSLPR signals

**Fig 2:**
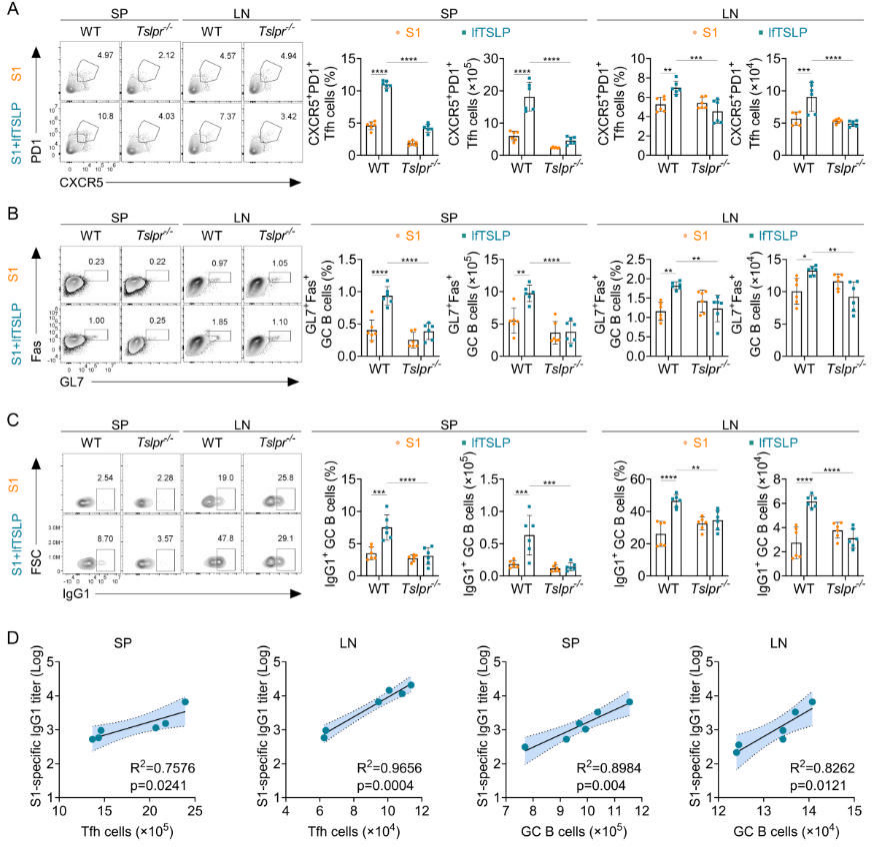
lfTSLP enhances Tfh cells and GC B cell responses after intranasal immunization. WT (*n* = 6) and *Tslpr^–/–^* mice (*n* = 6) were immunized by intranasal application of S1 alone or admixed with lfTSLP. Ten days after two booster immunizations, the spleen (SP) and LN were analyzed by flow cytometry for CXCR5^+^ PD-1^+^ Tfh cells among live CD4^+^ CD44^+^ cells (**A**), Fas^+^ GL7^+^ GC B cells among live CD19^+^ cells (**B**), and IgG1^+^ GC B cells among live CD19^+^ Fas^+^ GL7^+^ cells (**C**). (**D**) Correlation analysis between Tfh cell and GC B cell numbers of SP and LN and serum S1-specific IgG1 titers in S1 and lfTSLP-immunized mice 10 days after booster immunization. The Pearson correlation coefficient was used to determine the *r*-value for the correlation between the two groups. Results are representative of two independent experiments and shown as mean ± SEM. Each symbol represents the result of an individual animal. *P < 0.05, **P < 0.01, ***P < 0.001, ****P < 0.0001, by two-way ANOVA with Tukey’s multiple-comparison test (**A–C**).

T follicular helper (Tfh) cells are key regulators of germinal center (GC) B cell responses for the induction of high-affinity antibody production (28). We next characterized the frequencies of PD1^+^ CXCR5^+^ Tfh cells and GL7^+^ FAS^+^ GC B cells by flow cytometry in the spleen and mediastinal lymph nodes (LN) of WT and *Tslpr*^–/–^ mice immunized intranasally with 3 µg of S1 or supplemented with 4 µg of lfTSLP (Fig. 2). We found that intranasal immunization with the lfTSLP-enriched vaccine increased the proportion and number of Tfh cells in the spleen and LN of WT mice compared to *Tslpr*^–/–^ mice (Fig. 2A) or when the immunizations were given in the absence of lfTSLP (Fig. 2A). Similarly, the GC B cell response to vaccination with the lfTSLP-adjuvanted vaccine followed the same pattern as the Tfh cell responses, with an increased proportion and number of GC B cells in the spleen and LN of WT mice, which was then abolished in *Tslpr*^–/–^ mice (Fig. 2B). Moreover, WT mice immunized with the lfTSLP-adjuvanted vaccine had higher frequency and number of IgG1-producing GC B cells (IgG1^+^ GL7^+^ FAS^+^) in the spleen and LN than mice immunized with S1 alone, but the stimulatory effect of lfTSLP on the proportion of IgG1^+^ GC B cells was not augmented in *Tslpr*^–/–^ mice (Fig. 2C). A similar picture shows the induction of Tfh cell and GC B cell responses in the spleen and LN by lfTSLP when immunized with 5 µg of RBD antigen intranasally (Fig. S3A through C). However, such GC alterations were not induced by intranasal treatment of mice with the sfTSLP-formulated RBD vaccine (Fig. S3A through C) or the sfTSLP-formulated S1 vaccine (Fig. S4A through F). Intranasal lfTSLP treatment without vaccine failed to elicit the GC alterations observed under vaccination conditions in the spleen (Fig. S5A, C and E) and LN (Fig. S5B, D and F), indicating that antigen is required to drive the observed changes in immune cell composition. Interestingly, the numbers of Tfh cells and GC B cells from the spleen and LN were positively correlated with serum S1-specific IgG1 titers (Fig. 2D) and RBD-specific IgG1 titers (Fig. S3) in lfTSLP-complemented vaccine-immunized mice, indicating that lfTSLP-induced Tfh cell and GC B cell responses may contribute to the development of antibody responses after intranasal vaccination. Together, these data suggest that lfTSLP, but not sfTSLP, stimulates robust GC responses after mucosal immunization in a TSLP receptor-dependent manner, which correlates with vaccine antibody responses.

### lfTSLP boosts durable GC responses and humoral immunity

**Fig 3:**
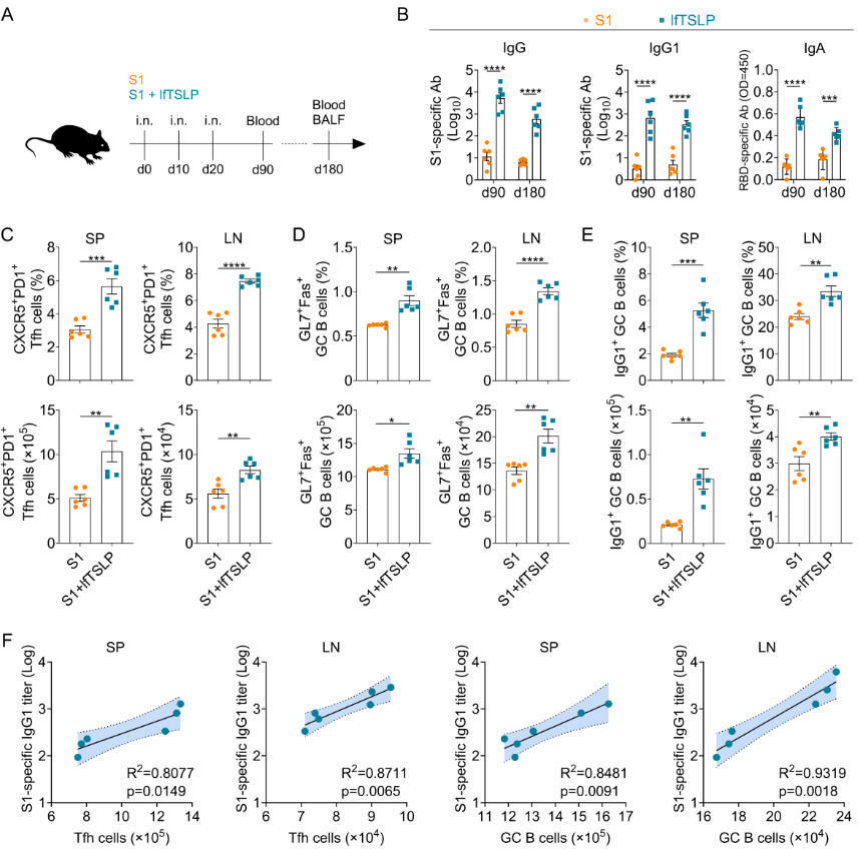
lfTSLP promotes long-lasting antibody and GC responses after intranasal immunization. (**A**) WT and *Tslpr^–/–^* mice immunized by intranasal application of S1 (*n* = 6) in the presence or absence of lfTSLP (*n* = 6) on days 0, 10, and 20. Serum was obtained on days 90 and 180, while the spleen (SP) and LN were harvested on day 180. (**B**) Serum S1-specific IgG and IgG1 titers and BALF S1-specific IgA titers were detected by ELISA. The percentages and numbers of CXCR5^+^ PD-1^+^ Tfh cells among live CD4^+^ CD44^+^ cells (**C**), Fas^+^ GL7^+^ GC B cells among live CD19^+^ cells (**D**), and IgG1^+^ GC B cells among CD19^+^ Fas^+^ GL7^+^ cells (**E**) in SP and LN were detected by FACS. (**F**) Correlation analysis between Tfh cell and GC B cell numbers of SP and LN, as well as serum S1-specific IgG1 titers, in mice immunized with S1 and lfTSLP after 180 days. The Pearson correlation coefficient was used to determine the r-value for the correlation between the two groups. Results are shown as mean ± SEM. *P < 0.05, **P < 0.01, ***P < 0.001, ****P < 0.0001, by two-way ANOVA with Tukey’s multiple-comparison test (**B**), unpaired two-tailed Student’s *t*-test (**C–E**), and two-tailed *t*-tests using Welch’s correction (**D**). Each symbol represents an individual animal.

To determine if antibody production and GC reactions were sustained by lfTSLP, mice were intranasally immunized with 3 µg of S1 or enriched with 4 µg of lfTSLP (Fig. 3A). Serum antibody levels were assessed at days 70 and 160 after the second booster vaccination, with significant S1-specific IgG and IgG1 titers still observed in serum from the lfTSLP-formulated S1 vaccination (Fig. 3B). Additionally, lfTSLP greatly increased the levels of S1-specific IgA levels in the BALF 70 and 160 days after the second booster vaccination (Fig. 3B). At day 160 after the second booster vaccination, the frequency and number of Tfh cells increased considerably in the spleen and LN of mice immunized in the presence of lfTSLP (Fig. 3C). Immunization with lfTSLP-enriched vaccines also elicited a higher proportion and number of GC B cells in the spleen and LN (Fig. 3D), which can constitutively produce high levels of IgG1 antibody (Fig. 3E). Importantly, the numbers of Tfh cells and GC B cells from spleen and LN were strongly linked with serum S1-specific IgG1 titers in lfTSLP-complemented vaccine-immunized animals (Fig. 3F). However, intranasal lfTSLP administration alone had no effect on such persistent GC reactions, suggesting that antigen co-delivery is required for lfTSLP-induced long-term GC response and humoral immunity (Fig. S5A through F). These results indicated that lfTSLP induces enduring GC responses and antibody production after mucosal immunization.

### The adaptive immune-enhancing effect of lfTSLP depends on cDCs

**Fig 4:**
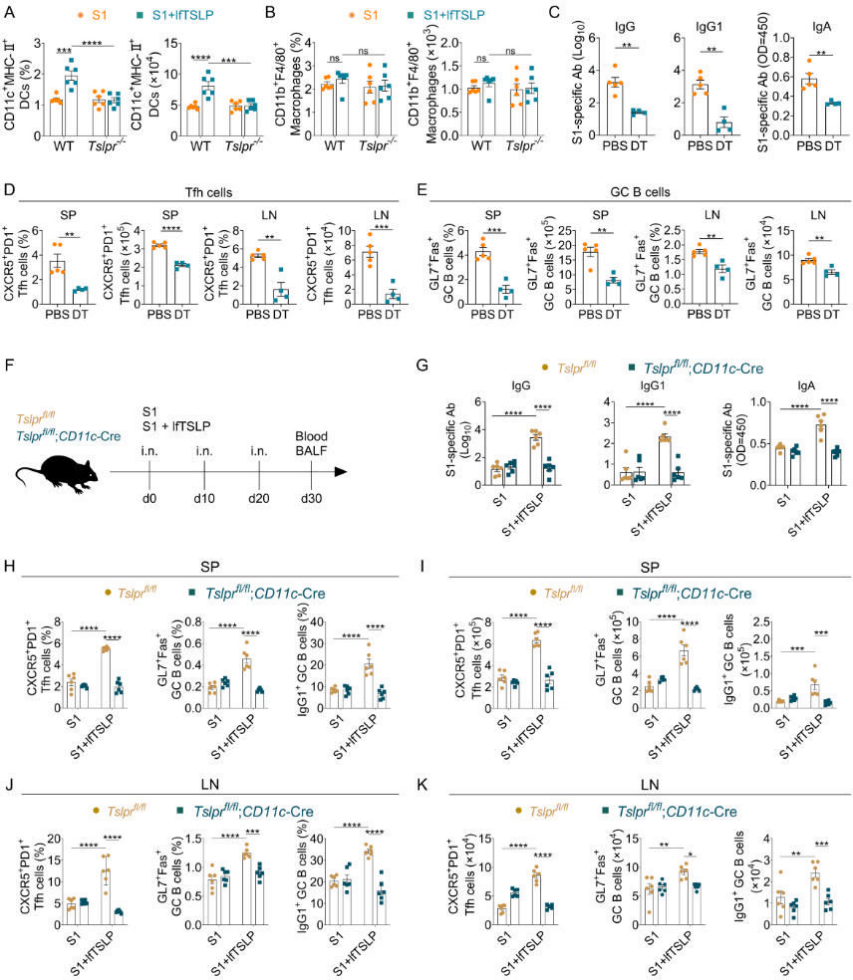
lfTSLP boosts CD11c^+^ cDC-dependent GC and antibody responses. (**A and B**) WT mice (*n* = 6) were intranasally immunized with S1 alone or combined with lfTSLP. On day 5 after post-boosting, the frequencies and numbers of cDCs (CD11c^+^ MHC-II^+^) (**A**) and macrophages (CD11b^+^ F4/80^+^) (**B**) in LN were determined by FACS. (**C–E**) WT (*n* = 6) and CD11c-DTR mice (*n* = 6) received intranasal vaccination with the S1 plus lfTSLP and were injected with diphtheria toxin (DT) or PBS on days 0, 10, and 20. (**C**) On day 30, S1-specific antibody titers in serum and BALF were detected by ELISA. (**D and E**) The frequencies and numbers of CXCR5^+^ PD-1^+^ Tfh cells among live CD4^+^ CD44^+^ cells and Fas^+^ GL7^+^ GC B cells among live CD19^+^ cells in SP and LN were detected by FACS. *n *= 4–5 mice per group. (**F**)* Tslpr^fl/fl^* (*n* = 6) and *Tslpr^fl/fl^;CD11c*-Cre mice (*n* = 6) were intranasally immunized with S1 in the presence or absence of lfTSLP. Serum and BALF were collected 10 days after the second booster immunization for detecting the titers of S1-specific antibodies, and the SP and LN were used for monitoring GC responses. (**G**) Serum and BALF S1-specific antibody titers were analyzed by ELISA. The percentages and numbers of CXCR5^+^ PD-1^+^ Tfh cells among live CD4^+^ CD44^+^ cells, Fas^+^ GL7^+^ GC B cells among live CD19^+^ cells, and IgG1^+^ GC B cells among CD19^+^ Fas^+^ GL7^+^ cells in SP (**H and I**) and LN (**J and K**) were measured by FACS. Results are representative of three independent experiments and shown as mean ± SEM. Each symbol represents an individual animal. **P < 0.01, **P < 0.01, ***P < 0.001, ****P < 0.0001, by two-way ANOVA with Tukey’s multiple-comparison test (**A and B, G–K**), two-tailed *t*-tests using Welch’s correction (**C**), and unpaired two-tailed Student’s *t*-test (**D, E**).

Conventional dendritic cells (cDCs) efficiently promote GC responses (29). To determine whether cDCs are the target cells of lfTSLP in inducing antibody production and GC responses in our system, we analyzed the number of cDC populations in intranasally immunized mice with 3 µg of S1 or formulated with 4 µg of lfTSLP. We observed an increase in the frequency and number of CD11c^+^ MHC-II^+^ cDCs in the LN of WT mice, but not *Tslpr*^–/–^ mice, that received the lfTSLP-adjuvanted S1 vaccines (Fig. 4A). Consistent with these findings, the number of CD11b^+^ F4/80^+^ macrophages was not altered in mice receiving lfTSLP-adjuvanted S1 vaccines (Fig. 4B), suggesting that lfTSLP might act on cDCs to exert adjuvant activity.

To examine whether lfTSLP-boosted adaptive responses depend on cDCs, we performed similar immunizations in CD11c-DTR (diphtheria toxin receptor) mice (Fig. 4C). During diphtheria toxin treatment, immunization of CD11c-DTR mice depleted of CD11c^+^ cDCs resulted in greatly reduced production of serum S1-specific IgG and IgG1, as well as BALF S1-specific IgA, in response to lfTSLP-formulated S1 immunization (Fig. 4C). Corresponding with reduced antibody responses, depletion of CD11c^+^ cDCs resulted in a significantly lower frequency and number of Tfh cells (Fig. 4D) and GC B cells (Fig. 4E) in the spleen and LN following intranasal immunization with the lfTSLP-formulated S1 vaccine. These results indicate that CD11c^+^ cDCs are necessary for lfTSLP-elicited powerful GC responses as well as robust antibody responses after mucosal immunization.

To further investigate whether TSLPR expression on DCs is required for lfTSLP-boosted adaptive immunity to mucosal immunization, we crossed conditional CD11c-Cre mice with floxed TSLPR (*Tslpr^fl/fl^*) mice to generate *Tslpr^fl/fl^;CD11c-*Cre mice for specific deletion of TSLPR on CD11c^+^ DCs. When these animals were intranasally inoculated with the lfTSLP-formulated S1 vaccine (Fig. 4F), S1-specific serum IgG, IgG1, and mucosal IgA levels were sustainably lower in *Tslpr^fl/fl^;CD11c-*Cre mice than *Tslpr^fl/fl^* mice (Fig. 4G). We also observed a decreased proportion and number of Tfh cells, GC B cells, and IgG1-producing GC B cells in the spleen (Fig. 4H and I) and LN (Fig. 4J and K) of *Tslpr^fl/fl^;CD11c-*Cre mice after intranasal immunization with the lfTSLP-formulated S1 vaccine. However, *Tslpr^fl/fl^* mice and *Tslpr*^*fl/fl*^;*CD11c-*Cre mice receiving S1 alone had equivalent IgG1 and IgA titers (Fig. 4G), as well as similar frequencies and numbers of Tfh cells and GC B cells in the spleen and LN (Fig. 4H through K). To verify the direct effects of lfTSLP on cDCs, we crossed CD19-Cre mice with floxed TSLPR (*Tslpr^fl/fl^*) mice to generate *Tslpr^fl/fl^;CD19-*Cre mice, which lack TSLPR on B cells for intranasal vaccination (Fig. S6A). As anticipated, the enhancing effects of lfTSLP in inducing antibody responses are identical between *Tslpr^fl/fl^;CD19-*Cre mice and *Tslpr^fl/fl^* mice (Fig. S6B), suggesting that TSLPR expression on B cells is dispensable for lfTSLP-induced antibody responses after intranasal immunization. Therefore, these results suggest that lfTSLP directly acts on cDCs, but not B cells, to improve GC responses and humoral immunity to mucosal immunization.

### Migratory cDC1 subtypes driven by lfTSLP improve humoral immunity

**Fig 5:**
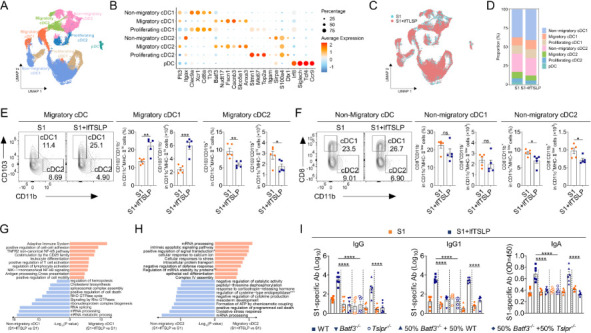
The immune-enhancing effect of lfTSLP depends on migratory cDC1s. WT mice were immunized with S1 in the presence or absence of lfTSLP intranasally, and CD11c^+^ DCs were purified on day 5 after the booster immunization for scRNA sequencing. *n *= 3 per group. (**A**) UMAP projection of 44087 CD11c^+^ DCs colored by different cell types. (**B**) Dot plot heatmap featuring marker genes for each cell type. The dot color represents the average expression of that gene within a cluster, and the dot size indicates the percentage of cells expressing the gene. (**C**) UMAP projection of 44087 DCs colored by S1 and S1 plus lfTSLP groups. (**D**) The proportion of DC subtypes in the S1 and S1 plus lfTSLP groups. (**E and F**) Representative FACS analyzing the percentages and numbers of migratory cDC1s (CD11c^+^ MHC-II^hi^ CD103^+^ CD11b^-^), migratory cDC2s (CD11c^+^ MHC-II^hi^ CD103^-^ CD11b^+^), non-migratory cDC1s (CD11c^+^ MHC-II^low^ CD11b^-^ CD8^+^), and non-migratory cDC2s (CD11c^+^ MHC-II^low^ CD11b^+^ CD8^-^) populations in LN. *n* = 5 mice per group. (**G and H**) The significantly enriched signatures in migratory or non-migratory cDC1s (**G**) and cDC2s (**H**) were compared from the S1 plus lfTSLP group to the S1 group. *n *= 3 mice per group. (**I**) The mixtures of bone marrow cells from the listed mouse strains were transplanted onto lethally irradiated WT mice. Six weeks later, the animals underwent intranasal application of S1 in the presence or absence of lfTSLP. Serum and BALF were analyzed for S1-specific antibodies by ELISA on day 10 after two booster immunizations. *n* = 6 mice per group. Results are shown as mean ± SEM. ns, no significant difference. *P < 0.05, **P < 0.01, ***P < 0.001, ****P < 0.0001, by unpaired two-tailed Student’s *t*-test (**E and F**) and one-way ANOVA with Tukey’s multiple-comparison test (**I**).

cDCs can be divided into two major subsets: type 1 conventional dendritic cells (cDC1s) and type 2 conventional dendritic cells (cDC2s) (30). Cell surface expression of CD103, CD8α, XCR1, or CLEC9A defines cDC1s, which rely on BATF3 and IRF8 during development (31, 32). In contrast, cDC2s were identified by surface expression of CD11b and Sirpα and require IRF4 signaling for development (31, 33). Recent studies in mice have revealed that cDCs are heterogeneous, with distinct phenotypic and functional features (34). To identify the full spectrum of cDC heterogeneity affected by lfTSLP in our system, we purified LN CD11c^+^ DCs from mice vaccinated with S1 in the presence or absence of lfTSLP for single-cell RNA sequencing (scRNA-seq). A total of 44,087 DCs were collected from three S1-immunized mice, and three lfTSLP-formulated S1-immunized mice were displayed on an unsupervised uniform manifold approximation and projection (UAMP) plot (Fig. 5A). We identified seven distinct DC clusters, namely non-migratory cDC1s (*Flt3^+^ Itagx^+^ Clec9a^+^ Xcr1^+^ CD8a^+^ Nudt17*^lo^), migratory cDC1s (*Flt3^+^ Baft3^+^ Fscn^+^ Cacnb3^+^ Slco5 a1^+^ Anxa3^+^ Nudt17*^hi^), proliferating cDC1s (*Flt3^+^ Xcr1^+^ Clec9a^+^ Cd8a^+^ Tlr3^+^ Stmn1^+^ Mki67^+^ Top2a^+^*), non-migratory cDC2s (*Flt3^+^ Itagx^+^ Itgam^+^ Sirpa^+^ S100 a4^+^ Dtx1^+^ Nudt17*^lo^), migratory cDC2s (*Flt3^+^ Baft3^+^ Fscn^+^ Cacnb3^+^ Slco5 a1^+^ Anxa3^+^ Nudt17*^hi^), proliferating cDC2s (*Flt3^+^ Itgax^+^ Stmn1^+^ S100 a4^+^ Dtx1^+^ Mki67^+^ Top2a^+^*), and plasmacytoid DCs (pDCs) (*Ifr8^+^ Singlech^+^ Tcl4^+^ Ccr9^+^*) (Fig. 5B, Fig. S7A and B), in agreement with recent scRNA-seq data that defined DC subtypes (34, 35). We observed that the proportion of cDC1s increased, whereas the proportion of cDC2s decreased, when mice were immunized with lfTSLP-adjuvanted S1 vaccines (Fig. 5C and D). To validate the influence of lfTSLP on cDC1s and cDC2s, we employed flow cytometry to investigate the alterations in their subpopulations in immunized mice (Fig. 5E and F). When compared with mice vaccinated with S1, animals immunized with S1, coupled with lfTSLP, had a considerably higher percentage and number of migratory cDC1s (CD11c^+^ MHC-II^hi^ CD103^+^ CD11b^-^) and a lower number of migratory cDC2s (CD11c^+^ MHC-II^hi^ CD103^-^ CD11b^+^) and non-migratory cDC2s (CD11c^+^ MHC-II^low^ CD11b^+^ CD8^-^) in the spleen and LN, while the ratio and number of non-migratory cDC1s (CD11c^+^ MHC-II^low^ CD11b^-^ CD8^+^) remained identical (Fig. 5E and F). This observation hints that migratory cDC1s may be active in response to lfTSLP signaling during vaccinations, which might be responsible for antibody responses.

To determine the potential functional effects of lfTSLP on migratory cDC1s, we performed enrichment analysis of the gene set for biological processes in gene ontology terms. The results indicated that antigen processing–cross-presentation, T cell activation, and adaptive immune system pathways were enriched in migratory cDC1s in mice that obtained lfTSLP-adjuvanted S1 vaccination versus S1 alone (Fig. 5G). In contrast, the RNA metabolic process, RNA splicing, and GTPase signaling pathways were enriched in the non-migratory cDC1s when mice received the lfTSLP-formulated S1 vaccination (Fig. 5G). Moreover, lfTSLP primarily governs mRNA processing, cellular stress, apoptosis, and negative regulatory signals in both migratory and non-migratory cDC2s (Fig. 5H). Migratory and non-migratory cDC1s and cDC2s exhibited distinct gene expression signatures involving antigen processing and presentation, cytokine and chemokine, cytokine and chemokine receptor, toll-like receptor activation, cell adhesion, and regulation expression in lfTSLP-formulated S1-immunized mice (Fig. S8A through D). Specifically, migratory cDC1s uniquely expressed elevated co-stimulatory molecules CD40 and ICOSL in lfTSLP-adjuvanted mice (Fig. S9A through E), which are known to regulate T cell-dependent humoral immune responses (36, 37). Gene set enrichment analysis (GSEA) showed that genes altered in migratory cDC1s from mice immunized in the presence of lfTSLP were associated with non-canonical NF-κB signaling pathways (Fig. S9F), which depend on the accumulation of NF-κB inducing kinase (NIK) and downstream p100 and p52 processing. lfTSLP treatment upregulated the expression levels of NIK, phosphor-100 (p-p100), p52, and RelB in migratory cDC1s from WT mice, but not in *Tslpr^−/−^* mice (Fig. S9G). However, NIK inhibitor treatment limits non-canonical NF-κB signaling activation by lfTSLP (Fig. S9H). Non-canonical NF-κB signaling is also required for lfTSLP-induced CD40 and ICOSL expression in migratory cDC1s, as revealed by administering lfTSLP-formulated S1-immunized mice with an NIK inhibitor (Fig. S9I). These results suggest that lfTSLP may influence migratory cDC1s to induce antibody production in our system.

*Batf3^−/−^* mice lack migratory cDC1s (38). When intranasal application of lfTSLP-formulated S1 vaccines was performed, chimeric mice reconstituted with bone marrow (BM) cells from *Batf3^−/−^* mice exhibited lower S1-specific serum IgG1 and BALF IgA levels than control mice (Fig. 5I), suggesting that migratory cDC1s are crucial in lfTSLP-enhanced antibody production. To investigate whether migratory cDC1s directly respond to lfTSLP for inducing antibody production, we constructed BM chimeric mice with TSLP receptor-deficient migratory cDC1s by regenerating lethally irradiated WT mice with a 1:1 ratio of BM cells from *Tslpr^−/−^* and *Batf3^−/−^* mice. Intranasal immunization with lfTSLP-adjuvanted S1 vaccines resulted in considerably reduced levels of S1-specific IgG1 and IgA in mixed BM chimeric mice compared to control mice with TSLP receptor-competent migratory cDC1s (Fig. 5I). These data revealed that lfTSLP promotes antibody production by acting on migratory cDC1s.

### The lfTSLP-adjuvanted vaccine confers cross-protection against SARS-CoV-2

**Fig 6:**
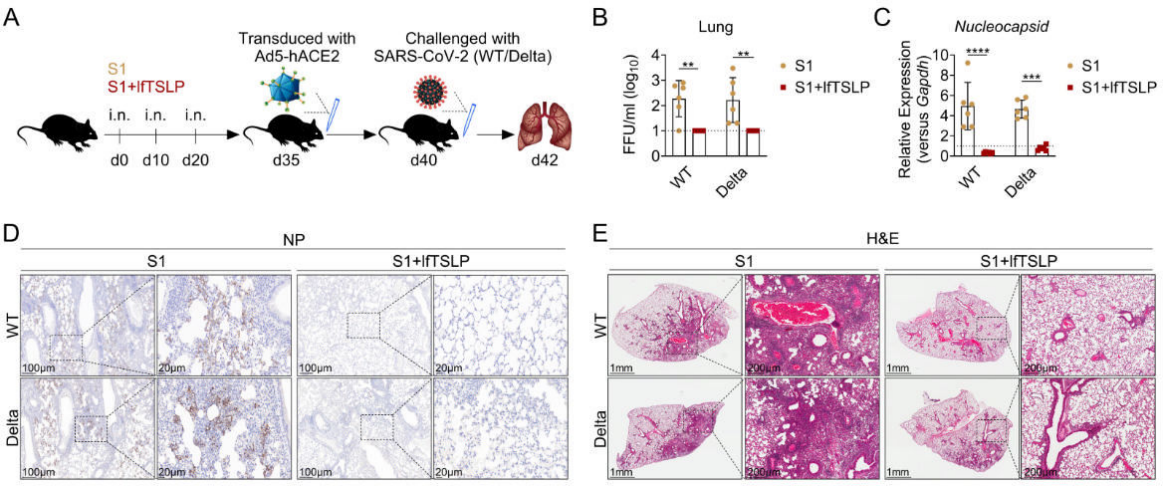
The lfTSLP-adjuvanted subunit vaccine provides cross-protection against SARS-CoV-2. (**A**) Mice were intranasally immunized with S1 in the presence or absence of lfTSLP on days 0, 10, and 20. On day 35, animals were transduced with Ad5-expressing hACE2 and then intranasally challenged with 5 ×  10^4^ FFU SARS-CoV-2 Wuhan (WT) or 5 ×  10^4^ FFU B.1.617 (Delta) strains on day 40. *n* = 6 mice per group. Viral titration (**B**), viral nucleocapsid RNA (**C**), and viral nucleocapsid protein (NP) (**D**) analysis in the lungs, and the lung tissues were harvested on day 42 for H&E staining (**E**). Results are shown as mean ± SEM. **P < 0.01, ***P < 0.01, ****P < 0.0001, by two-way ANOVA with Tukey’s multiple-comparison test (**B and C**).

To determine the biological implications of lfTSLP-boosted vaccine immune enhancement, mice were intranasally immunized with S1 three times at 10-day intervals, with or without lfTSLP. Mice were intranasally challenged with 5 × 10^4^ FFU SARS-CoV-2 wild-type (WT) or 5 × 10^4^ FFU B.1.617.2 (Delta) strains after being transduced with Ad5-expressing hACE2, facilitating ectopic expression of hACE2 for effective SARS-CoV-2 infection (Fig. 6A). To assess viral replication in the lungs at day 2 post-infection, all S1-immunized animals showed very high viral load (Fig. 6B), viral RNA (Fig. 6C), and viral nucleocapsid protein levels (Fig. 6D) of WT and Delta strains. Surprisingly, all mice in the lfTSLP-complemented S1 vaccination group were entirely protected from WT and Delta infection (Fig. 6B through D). Furthermore, histological analysis of lungs from the lfTSLP-complemented S1-immunized mice revealed considerably lower lung pathology than lfTSLP-free vaccine-immunized mice at day 2 post-infection of WT and Delta strains (Fig. 6E). These findings demonstrated that mucosal vaccines adjuvanted with lfTSLP protect mice against SARS-CoV-2 WT and Delta strains.

## DISCUSSION

Our work illuminates previously unidentified differences between two human TSLP variants in modulating vaccine-mediated adaptive immunity by head-to-head comparison, demonstrating that lfTSLP rather than sfTSLP enhances durable antigen-specific systemic and mucosal antibody synthesis after mucosal vaccination with commercial SARS-CoV-2 subunit vaccines, resulting in improved resistance to infection with SARS-CoV-2 wild-type and Delta variants. The mucosal adjuvant activity of lfTSLP is mechanistically ascribed to targeted migratory cDC1 subtypes, which augments sustained GC responses. Our findings indicate that lfTSLP functions as a potent mucosal adjuvant, orchestrating systemic and mucosal adaptive immunity through migratory cDC1-dependent mechanisms.

Among the two human TSLP isoforms, lfTSLP has been extensively researched and is regarded as a central regulator of type 2 helper T cell (Th2) responses, but the biological role of sfTSLP is largely unknown (8, 9). Recent studies have reported distinctions in the human TSLP isoforms, with lfTSLP promoting inflammatory responses and slTSLP inhibiting inflammatory responses (8, 9). Our finding surprisingly revealed that lfTSLP enhances the systemic and mucosal adaptive immune responses during mucosal vaccination, but not when immunization is supplemented with sfTSLP. Importantly, *Tslpr^−/−^* mice are unable to induce antibody production when subunit vaccines are enriched with lfTSLP. Our observations implied that the unique features of lfTSLP as a potent mucosal adjuvant depend on TSLP receptor (TSLPR) signaling and cannot be mimicked by sfTSLP. However, it remains unclear whether the difference in adjuvant activity between lfTSLP and sfTSLP is a result of their interaction with TSLPR. Previous studies indicate that lfTSLP has four α-helices (αA, αB, αC, and αD) linked by a BC loop and two long overhand AB and CD loop regions, whereas sfTSLP only covers the αC, αD, and the long CD loop of lfTSLP (39). Since αA, αB, and AB loops have a crucial role in TSLP-TSLPR binding and IL-7Rα recruitment (39), it is unclear whether sfTSLP fails to induce mucosal adaptive immunity due to a lack of these regions. Structural and molecular biology approaches might shed light on this conundrum.

Many types of immune cells express the TSLP receptor (9). We observed that depleting CD11c^+^ DCs *in vivo* abolished the potential of lfTSLP to enhance antigen-specific antibody production as well as promote Tfh cell and GC B responses in the spleen and lymph nodes, indicating that cDCs are essential for lfTSLP-induced humoral immunity. In addition, deleting TSLPR in cDCs, but not B cells, impairs the immune-enhancing functions of lfTSLP in inducing Tfh cells and GC B cell responses, thereby diminishing antigen-specific antibody production. Thus, lfTSLP might enhance antigen presentation by cDCs, culminating in improved antigen-specified humoral immunity. In general, cDCs can be divided into two major subsets: cDC1s and cDC2s (30). Although both cDC1s and cDC2s have functional roles for Tfh cells, an increasing number of recent studies have revealed the high heterogeneity and functional characteristics of human and mouse cDC subtypes (29, 34). Which cDC subpopulations respond to lfTSLP adjuvant responses in mice? To answer this question, we applied scRNA-seq analysis and strikingly identified that migratory cDC1s are abundant in mice with lfTSLP-adjuvanted vaccines. In contrast, other DC subtypes, such as cDC2s and pDCs, were significantly reduced in mice with lfTSLP-adjuvanted vaccines compared to non-adjuvanted vaccines. Furthermore, *Baft3^−/−^* mice, which lack cDC1s, showed attenuated immunoenhancement activity of lfTSLP after intranasal immunization. Although Baft3-deficient mice also lack non-migratory cDC1s, our observations indicated that lfTSLP did not affect the number of non-migratory cDC1s, and antigen processing-cross (APC) presentation and T cell activation signaling pathways were highly enriched in migratory cDC1s rather than non-migratory cDC1s in response to lfTSLP administration. Employing mixed bone marrow chimeric mice, we provide unequivocal evidence demonstrating that lfTSLP must act on migratory cDC1s to exhibit an immune-enhancing impact.

Our data demonstrate that lfTSLP upregulates the expression of costimulatory molecules CD40 and ICOSL in migratory cDC1s. The promoter of CD40 and ICOSL associates with the non-canonical NF-κB complex (40, 41), and non-canonical NF-κB signaling blockade in migratory cDC1s significantly inhibits the enhancing effects of lfTSLP on CD40 and ICOSL. These findings reveal an undefined molecular mechanism by which migratory cDC1s promote lfTSLP adjuvant responses. Previous *in vitro* cell co-culture experiments revealed that human TSLP can activate human blood primary DCs to drive human Tfh cell differentiation and IgE production through the co-stimulatory molecule OX40L (42). Human TSLP is also reported to amplify human epithelial cell-induced IgG and IgA class switching by stimulating monocyte-derived myeloid DCs to produce more BAFF (43), showing that human TSLP exerts multiple immune regulatory functions in distinct cDC1 subtypes. Our observations in mice extend the understanding of the immunomodulatory properties of lfTSLP as a mucosal adjuvant and further underline the importance of non-canonical NF-κB signaling of lfTSLP in the activation of migratory cDC1s. Notably, CD40-CD40L and ICOSL-ICOS interactions license DCs to drive Tfh cell activation and differentiation and facilitate Tfh cell-mediated B cell activation (36, 37). However, whether lfTSLP depends on CD40 and ICOSL on migratory cDC1s to enhance GC responses and humoral immunity requires further elucidation.

Our results highlight the effective adjuvant effects of lfTSLP when combined with commercial SARS-CoV-2 subunit vaccines after intranasal immunization. Fragments of the spike (S) protein, such as S1 or RBD, have been identified as promising vaccine candidates against SARS-CoV-2 (3, 5, 44, 45). The durability of vaccine-induced immune responses is crucial for providing long-term protection against SARS-CoV-2 infection (46, 47). Our findings demonstrated that intranasal immunization with the S1 or RBD vaccine enriched with lfTSLP robustly induced systemic and mucosal antibodies that remained elevated for several months as well as sustained GC reactions. Additionally, through intranasal immunization with lfTSLP, substantial amounts of neutralizing antibodies against the SARS-CoV-2 WT strain occur. The emergence of SARS-CoV-2 variants, such as Delta, Omicron, and its subvariants, has raised concern that these variants would evade the neutralizing antibodies produced in response to vaccination (48–51). Surprisingly, after intranasal immunization with lfTSLP-adjuvanted vaccines, mice maintained substantial levels of S1-specific neutralizing antibodies against the Delta strain. More importantly, virus challenge studies in mice showed that lfTSLP-boosted mucosal adaptive immunity confers cross-protection against SARS-CoV-2 WT and Delta strains, suggesting that lfTSLP can enhance vaccine-induced antiviral immunity.

In conclusion, our work in mouse models highlights an early unrecognized distinct immunomodulatory role of two human TSLP variants, with lfTSLP, but not sfTSLP, playing a decisive role in vaccine-mediated adaptive protective immune responses as a potent mucosal adjuvant. We demonstrated that the boosting effects of lfTSLP on the adaptive immune systems are dependent on targeting migratory cDC1 subtypes to improve GC responses. Intranasal vaccine administration with a combination of the lfTSLP adjuvant elicited robust mucosal vaccine effectiveness, implying that it might be a promising platform for protection against both disease and infection and thus warrants further clinical evaluation in humans.

## MATERIALS AND METHODS

### Mice, viruses, and cells

C57BL/6 mice (designated WT) were purchased from the Guangdong Medical Laboratory Animal Center (Guangdong, China). *Tslpr^–/–^* mice were purchased from GemPharmatech Co., Ltd. (Nanjing, China). CD11c-DTR mice were the kind gift of Professor Fei Li (Fudan University, Shanghai, China), and *Baft3^–/–^* mice were the kind gift of Professor Jun Chen (Sun Yat-sen University, Guangdong, China).

To generate dendritic cell-specific *Tslpr* knockout mice, *CD11c*-Cre mice were purchased from Janvier Laboratories, and *Tslpr^fl/fl^* mice were purchased from GemPharmatech Co., Ltd. (Nanjing, China). Dendritic cell-specific *Tslpr* knockout mice (designated *Tslpr^fl/fl^;CD11c*-Cre) were generated by crossbreeding *CD11c*-Cre mice with *Tslpr^fl/fl^* mice and confirmed by tail genotyping. Littermate *Tslpr^fl/fl^* mice were used as controls.

To generate B cell–specific *Tslpr* knockout mice, *Tslpr^fl/fl^* mice and *CD19*-Cre mice were purchased from GemPharmatech Co., Ltd. (Nanjing, China). *CD19*-Cre mice were bred with *Tslpr^fl/fl^* mice to obtain B cell–specific *Tslpr* knockout mice (designated *Tslpr^fl/fl^;CD19*-Cre). Littermate *Tslpr^fl/fl^* mice were used as controls.

DCs and Vero E6 cells were cultured in RPMI 1640 medium (Gibco, 11875500BT) supplemented with 10% fetal bovine serum (FBS). HEK293T cells were grown in DMEM medium (Gibco, 11995065) supplemented with 10% FBS. The SARS-CoV-2 strains and the human serotype 5 adenoviral vector expressing human ACE2 under the control of the CMV promoter were previously described (52).

### Bone marrow-derived DC generation and stimulation

Bone marrow cells were isolated from WT and *Tslpr^–/–^* mice, depleted of red blood cells, and cultured in RPMI 1640 medium supplemented with 50 ng/mL GM-CSF (Peprotech, AF-315-03) and 50 ng/mL IL-4 (Peprotech, AF-214-14) at 37°C and 5.0% CO_2_ for seven days. Bone marrow-derived DCs (BMDCs) were purified using a CD11c magnetic cell-sorting kit (Miltenyi Biotec, 130-125-835) according to the manufacturer’s instructions. After purification, BMDCs were cultured in RPMI 1640 medium without GM-CSF and IL-4 for five days before being stimulated with sfTSLP (100 ng/mL), lfTSLP (100 ng/mL), or mouse TSLP (Novoprotein, CJ69) for five or 15 minutes, respectively, and then the expression of phospho-STAT5 and STAT5 was analyzed by Western blotting.

### Immunization procedures

WT, *Tslpr^–/–^*, *Tslpr^fl/fl^*, *Tslpr^fl/fl^;CD11c*-Cre, and *Tslpr^fl/fl^;CD19*-Cre mice were intranasally immunized with 30 μL of 3 μg commercial SARS-CoV-2 (Wuhan strain) S1 protein (Genscript, Z03501) or 5 μg commercial SARS-CoV-2 (Wuhan strain) RBD protein (Genscript, Z03483) in the presence or absence of either 4 μg sfTSLP (63 amino acids [MFAMKTKAALAIWCPGYSETQINATQAMKKRRKRKVTTNKCLEQVSQLQGLWRRFNRPLLKQQ] were synthesized by ChinaPeptides, China) or 4 μg lfTSLP (Novoprotein, CK16). Immunizations were performed three or four times with 10-day intervals after anesthetization with isoflurane. Some animals were treated with either DMSO (vehicle, Solarbio, D8371) or NIK inhibitor (αNIK, MCE, HY-112433). Serum and bronchoalveolar lavage fluid (BALF) were collected after the second or third booster immunization to analyze S1- or RBD-specific antibodies by ELISA. Spleen and mediastinal lymph nodes (defined as LN) were collected for subsequent experiments. In some experiments, WT mice were intranasally treated with 4 μg sfTSLP or 4 μg lfTSLP alone three times with 10-day intervals following anesthetization with isoflurane. The spleen and LN were harvested on days 30 and 180 for further research.

For CD11c-DTR mouse immunization, mice were injected intraperitoneally with 5 ng/g body weight diphtheria toxin (DT, Sigma-Aldrich) on days 0, 10, and 20 to deplete DC *in vivo*. Control mice were administered an equivalent volume of PBS. On the day of DT injection, these animals were anesthetized with isoflurane and intranasally immunized with 3 μg SARS-CoV-2 (Wuhan strain) S1 protein in the presence of 4 μg lfTSLP. On day 10, following the second booster immunization, serum and BALF were obtained for ELISA analysis of S1-specific antibodies, while the spleen and LN were collected for FACS analysis of Tfh cells and GC B cells.

### Bone marrow chimeras

To generate mixed chimeric mice with TSLPR-deficient migratory cDC1s, WT recipient mice were lethally irradiated (2  ×  5.5 Gy, 4-hour interval) and reconstituted with 10^7^ bone marrow cells from 100% *Tslpr^–/–^* mice, 100% *Baft3^–/–^* mice, or mixes of 50% *Tslpr^–/–^* mice and 50% *Baft3^–/–^* mice. Mice were allowed to reconstitute for six weeks before intranasal immunization with 3 μg of SARS-CoV-2 (Wuhan strain) S1 in the presence or absence of 4 μg of lfTSLP three times, 10 days apart. Blood and BALF samples were taken on day 10 following the second booster immunization.

### Preparation of bronchoalveolar lavage fluids (BALF)

Bronchoalveolar lavage fluids (BALF) were acquired by inflating the lungs with 500 μL of PBS via a peripheral venous catheter (BD Venflon) and lavaging three times. BALF was centrifuged at 2,000 rpm for 4 minutes. The BAL fluid supernatants were kept at −80°C and then analyzed for IgA levels.

### Migratory cDC1 preparation and stimulation

Migratory cDC1s (CD11c^+^ MHC^hi^ CD103^+^ CD11b^-^) were sorted from LN with a pool of 10–15 WT and Tslpr^–/–^ mice using a BD FACSAria Special Order Cell Sorter (BD Biosciences). Sorted migratory cDC1s were cultured with RPIM1640 (Gibco, C11875500BT) supplemented with 10% fetal bovine serum (FBS, Procell, 164210-500) and 1% penicillin-streptomycin (P/S, Transgene, FG101-01) and treated with either DMSO (vehicle, Solarbio, D8371) or 10 µM NIK inhibitor (αNIK, MCE, HY-112433) for 24 hours in the presence or absence of lfTSLP (100 ng/mL) for 1 hour or 24 hours. Non-canonical NF-κB-pathway-related molecules were then analyzed by Western blot.

### Single-cell RNA sequencing and data analysis

Mice were intranasally immunized with 30 µL of 3 µg SARS-CoV-2 S1 in the presence or absence of 4 µg lfTSLP two times at 10-day intervals after anesthesia with isoflurane. On day 5 after booster immunization, fresh LNs were immediately resected and ground with a tissue homogenizer before being resuspended in PBS with 1% FBS (Procell, 164210-500) to prepare a single-cell suspension. After that, cells were passed through a 70 μm filter and processed for CD11c^+^ DCs enrichment by using the Dynabeads Mouse DC Enrichment Kit (Invitrogen, 114290) and then immediately resuspended in 1 mL of cold PBS with 2% FBS for scRNA-seq library preparation. Single-cell suspensions were processed using the 10 Genomics Single Cell 3’ Library Construction Kit v3. Approximately 2.6 × 10^4^ cells per chip run were loaded, generating gel beads-in-emulsions (GEMs), followed by reverse transcription, cDNA amplification, and library construction. Library concentration and product size were assessed using Qubit and Qsep100 before sequencing, respectively. The library sequencing was performed on the Illumina NovaSeq6000 instrument with paired-end 150 bp reads. The library structure comprised 28 bp Read 1, 8 bp 17 Index, 8 bp 17 Index, and 91 bp Read2.

Cell Ranger 5.0.1 (10x Genomics) was used for demultiplexing, read mapping to the reference genome (refdata-gex-mm10-2020-A), cell barcode and UMI correction, quantification of expression, and generation of the single-cell expression matrix. The resulting filtered matrix was loaded into Seurat 5.0 for subsequent analysis (53).

#### Quality control

A series of quality control steps were implemented on the filtered data. The expression matrix was loaded, and the top 50 PCs were used to do cell clustering. Initially, we excluded clusters exhibiting a median number of detected genes below 200. Subsequently, individual cells with detected genes below 200 or a mitochondrial gene percentage exceeding 20% were also filtered out. Doublet detection was performed using Scrublet with default settings (54). The observed bimodal distribution of doublet scores prompted the selection of the lowest point between the two peaks as the threshold, with cells exceeding this threshold classified as doublets. Following doublet removal, the final expression matrices from all samples were consolidated for downstream analyses.

#### Clustering

The merged matrix underwent normalization using the *LogNormalize* algorithm. The identification of 3,000 highly variable genes was performed using the *VST* algorithm. Gene expression was standardized using the z-score transformation. PCA analysis was employed for unsupervised linear dimensionality reduction of the data. The top 50 PCs, 30 perplexities, and 1.2 resolutions were used to identify cell clusters through the shared nearest neighbor (SNN) algorithm. For fine-grained analysis, all DCs were extracted, and their sub-clusters were identified using the top 40 PCs, 30 perplexity, and 2.0 resolution.

#### Data visualization

Building on the initial PCA-based dimensionality reduction, we further employed Uniform Manifold Approximation and Projection (UMAP) for non-linear projection of the data (55, 56). UMAP boasts exceptional computational efficiency, enabling rapid navigation of complex cellular landscapes. We employed the same PCs used for cell clustering as input for UMAP, ensuring alignment with the initial dimensional reduction and facilitating detailed exploration of fine-grained cellular subpopulations.

#### Identification of differentially expressed genes

To identify differentially expressed genes (DEGs) between the two cell groups, we implemented a two-pronged approach. For broad comparisons, we utilized either the FindAllMarkers or FindMarkers functions in Seurat. Both tools employ the two-sided Wilcoxon test to identify genes differentially expressed between groups. To manage the potential for false positives with multiple comparisons, both tools employ the Bonferroni correction for adjusted *P*-values. Additionally, for targeted exploration of cluster-specific gene expression, we leveraged the R package dplyr to efficiently extract genes uniquely expressed within each identified cluster.

#### GO enrichment analysis for differentially expressed genes

GO enrichment analysis of DEGs was performed using Metascape (https://metascape.org/), a user-friendly web-based platform that integrates data from multiple databases. The results were visualized as bar plots using the R package ggplot2.

#### Gene set enrichment analysis (GSEA)

The R package fgsea was employed for GSEA on specific signaling pathways. Firstly, the DEGs of migratory cDC1s between the S1 plus lfTSLP and the S1 groups were extracted and ranked based on log_2_ (FC) values. Then, the gene names were converted from SYMBOL to ENTREZID using the bitr function within the clusterProfiler package (57). Signaling pathways from the reactome.db database were subjected to enrichment analysis using the fgsea function. Finally, results were visualized using the gggsea and ggplot2 R packages.

### ELISA

To determine SARS-CoV-2 S1- or RBD-specific antibodies in serum and BALF, 96-well plates (Thermo Fisher, 446469) were coated with S1 or RBD protein at 4°C overnight. The plates were washed five times with PBST (PBS with 0.05% Tween-20 [A100777, Sangon]) and then blocked for 2 hours with 5% BSA (Solarbio, A8020) in PBS. Diluted serum samples were incubated at room temperature (RT) for 1 hour. The binding antibodies were then detected using horseradish peroxidase (HRP)-conjugated antibodies against IgG (Invitrogen, 626520), IgG1 (Invitrogen, A10551), IgG2c (Abcam, ab97255), or IgA (Invitrogen, 626720). The plates were incubated with a tetramethylbenzidine (TMB) substrate (Transgen, H101-01) for 10–30 minutes, and the reaction was then terminated by adding 1 M HCL, and absorbance was measured at 450 nm using a SpectraMax 340PC Microplate Reader (Molecular Devices, USA). ELISA endpoint titers were defined as the highest dilutions that result in OD values that were twofold higher than background.

### Flow cytometry

Single-cell suspensions of the spleen and LN were incubated with anti-mouse CD16/32 (BioLegend, 93) for 30 minutes on ice to block non-specific antibody binding. Dead cells were gated out by staining with LIVE/DEAD fixable near-IR dead cell dye (Invitrogen). For staining DCs, Tfh cells, and GC B cells, cell surface markers were stained with mAbs against CD11c (BioLegend, N418), MHC-II (BioLegend, M5/114.15.2), F4/80 (BioLegend, BM8), CD103 (BioLegend, 2E7), CD11b (BioLegend, M1/70), CD8a (BioLegend, 53-6.7), CD4 (BioLegend, GK1.5), CD44 (BioLegend, IM7), CXCR5 (BioLegend, L138D7), PD1 (BioLegend, 29F.1A12), CD19 (BioLegend, 6D5; MB19-1), GL7 (BioLegend, GL7), and/or FAS (BioLegend, SA367H8). For intracellular staining, cells were fixed and permeabilized with Cytofix/Cytoperm (BD Biosciences, 554714) according to the manufacturer’s instructions, and cells were incubated with an anti-mouse IgG1 antibody (Biolegend, RMG1-1). For staining co-stimulatory molecules on migratory cDC1, cells were stained with mAbs against CD40 (Biolegend, 3/23) and ICOSL (Biolegend, HK5.3). Cells were washed and fixed with 1% PFA for 30 minutes prior to acquisition. All samples were acquired on a NovoCyte Advanteon (Agilent), and data were analyzed in FlowJo software (Treestar).

Tfh cell gating strategy: cells were gated based on forward and side scatters, single cells, and live cells, and subsequently, CXCR5^+^ PD-1^+^ Tfh cells were gated based on CD4, CD44, CXCR5, and PD-1 expression. GC B cell gating strategy: cells were gated by forward and side scatters, single cells, and live cells, and subsequently, Fas^+^ GL7^+^ GC B cells were gated based on CD19, Fas, and GL7 expression. IgG1^+^ GC B cells were gated based on CD19, Fas, GL7, and IgG1 expression. DC subpopulation gating strategy: cells were gated based on forward and side scatters, single cells, and live cells, and then, DCs were gated based on CD11c and MHC-II expression. Migratory cDC1s were gated based on CD11c, MHC-II (high), CD103, and CD11b (negative) expression. Migratory cDC2s were gated based on CD11c, MHC-II (high), CD103 (negative), and CD11b expression. Non-migratory cDC1s were gated based on CD11c, MHC-II (low), CD11b (negative), and CD8 expression. Non-migratory cDC2s were gated based on CD11c, MHC-II (low), CD11b, and CD8 (negative) expression. Macrophage subpopulation gating strategy: cells were gated based on forward and side scatters, single cells, and live cells, and then macrophages were gated on CD11b and F4/80 expression.

### Western blotting

Migratory cDC1s were harvested and lysed in RIPA buffer (Solarbio, R0010) supplemented with protease inhibitor cocktail (MCE, HY-K0010) and phosphatase inhibitor cocktail I/II (MCE, HY-K0021, HY-K0022), and supernatants were collected. Lysates were kept on ice for 10 minutes before being ultrasonically processed for 30 seconds, five times, at 30-second intervals. Then, it was centrifuged at 12,000 rpm for 10 minutes. The supernatants were boiled at 95°C for 5 minutes with loading buffer (Solarbio, P1040) and resolved by SDS-PAGE gels (Beyotime, P0012AC). SDS-PAGE-resolved proteins were transferred to a PVDF membrane (Millipore, ISEQ00010) using a Western blotting system (Guangzhou Daoyi, Basic 400). Membranes were blocked with 5% skim milk and incubated with the primary antibodies NIK (Cell Signaling, 4994), p100 (Cell Signaling, 4882), phospho-p100 (Cell Signaling, 4810), RelB (Cell Signaling, 4922), STAT5 (Proteintech, 13179-1-AP), phospho-STAT5 (Cell Signaling Technology, 9351), and β-actin (Abcam, ab213262) at 4°C overnight. The membranes were washed in PBST and incubated with HRP-conjugated anti-rabbit secondary antibody (Cell Signaling, 7074S) for one hour at RT, before blots were developed on film using Meilunbio fg Super Sensitive ECL Luminescence Reagent (Meilunbil, MA0186).

### RT-qPCR

Lungs were homogenized with beads using a homogenizer (Lawson, China), and total RNA was extracted with Trizol (Takara, 9109) according to the manufacturer’s instructions and then reverse-transcribed into cDNA using the Thermoscript RT-PCR system according to the manufacturer’s instructions (Invitrogen, K1621). Quantitative real-time PCR (RT-qPCR) was run on the Bio-Rad sequence detection system with the SYBR Green Master mix (Applied TransGen Biotech, AQ1141-04) using primers against the SARS-CoV-2 nucleocapsid gene (forward primer: 5′-ATGCTGCAATCGTGCTACAA-3′, reverse primer: 5′-GACTGCCGCCTCTGCTC-3′) and Gapdh (forward primer: 5′-CTCTCTGCTCCTCCCTGT-3′, reverse primer: 5′-GCAACAATCTCCACTTTG-3′). Relative mRNA expression was calculated by the 2^-ΔCt^ according to Gapdh gene abundance.

### SARS-CoV-2 challenge

Mice were intranasally immunized with 30 μL of 3 μg commercial SARS-CoV-2 Wuhan (WT strain) S1 protein in the presence or absence of 4 μg lfTSLP, three times with 10-day intervals following anesthesia with isoflurane. Animals were transduced with 2.5 × 10^8^ FFU of adenoviral vectors expressing hACE2 (Ad5-hACE2). Five days post-transduction, mice were infected intranasally with 5 × 10^4^ FFU of the SARS-CoV-2 Wuhan (WT) strain or the B.1.617 (Delta) strain in a total volume of 50 μL of DMEM. All work with SARS-CoV-2 was conducted in the Biosafety Level 3 (BSL3) laboratories of the Guangzhou Customs District Technology Center. Two days post-infection, viral titers and viral nucleocapsid gene expression in lung tissues were determined by plaque assay and RT-qPCR. While some lung tissues were fixed for histopathology analyses and viral nucleocapsid protein, blood was collected on day 10 after two booster immunizations for SARS-CoV-2 neutralization assays.

### SARS-CoV-2 neutralization assay

For the SARS-CoV-2 pseudotyped virus neutralization assay, serum from immunized mice was diluted by DMEM (Gibco, 11995065) with 10% FBS and 1× penicillin-streptomycin (P/S), and then preincubated with GFP-luciferase pseudoviruses in 96-well plates for 6 hours, including SARS-CoV-2 Wuhan WT (Genomeditech, GM-0220PV07) and Delta B.1.617.2 (Genomeditech, GM-0220PV45) pseudotyped viruses. Then, the hACE2-expressing HEK293T cells (Abclonal, RM02456) were added at a density of 2 × 10^4^ cells/well and incubated for an additional 48 hours to allow infection. The luciferase reactions were carried out using a Single Luciferase Report Assay Kit (TransGen, FR101), and relative light units (RLUs) were detected with a SpectraMax Gemini XPS Microplate Reader (Molecular Devices, USA). IC_50_ was further analyzed using four-parameter logistic regression.

For the SARS-CoV-2 authentic virus neutralization assay, serum from vaccinated mice was serially diluted and incubated with 10^2^ FFU of the SARS-CoV-2 WT strain or Delta B.1.617 strain for 1 hour at 37°C. The mixture was then added to Vero E6 cells in 96-well plates and incubated for 1 hour. The inoculums were removed before adding 100 μL of 1.6% carboxymethylcellulose warmed to 37°C per well. The plates were then incubated at 37°C for 24 hours. Overlays were removed, and cells were fixed in 4% paraformaldehyde before being permeabilized with 0.2% Triton X-100. Cells were subsequently incubated with a rabbit anti-SARS-CoV-2 nucleocapsid protein (NP) polyclonal antibody (Sino Biological, 40143-T62). Next, HRP-labeled goat anti-rabbit secondary antibody (Jackson ImmunoResearch Laboratories, 111-035-144) was used. The reactivation was developed with TrueBlue Peroxidase Substrate (KPL, Gaithersburg, USA) and counted with an ELISPOT reader (Cellular Technology Limited, Cleveland, USA). IC_50_ was determined using four-parameter logistic regression.

### Focus-forming assay (FFA)

Viral titers were determined by the FFU assay. Vero E6 cells (CRL-1586, ATCC) were seeded in 96-well plates one day before infection. Lung tissues were homogenized in 1 mL of sterile PBS using the MagNA lyser (Roche) and centrifuged for 10 min at 8,000 rpm. Then, the resulting supernatants were serially diluted and utilized to inoculate cells at 37°C for 1 hour. The inocula were then removed before adding 100 μL of 1.6% carboxymethylcellulose, warmed to 37°C, per well. After 24 hours, cells were fixed with 4% paraformaldehyde and permeabilized with 0.2% Triton X-100. Cells were then incubated with a rabbit anti-SARS-CoV-2 nucleocapsid protein (NP) polyclonal antibody (Sino Biological, 40143-T62) and then followed by HRP-labeled goat anti-rabbit secondary antibody (Jackson ImmunoResearch Laboratories, 111-035-144). The foci were visualized by TrueBlue Peroxidase Substrate (KPL, Gaithersburg, USA) and counted with an ELISPOT reader (Cellular Technology Limited, Cleveland, USA).

### Lung pathology

To examine the lung pathology, the lungs were removed from mice in each group and fixed in 4% paraformaldehyde (Biosharp, BL539A) for more than 24 hours. Then, the lungs were embedded in paraffin, sectioned into four-micron thickness, and stained with hematoxylin-eosin (H&E) (Servicebio, G1076), according to the manufacturer’s instructions. Stained slices were scanned with an upright white-light photography microscope (Nikon, Eclipse E100).

### Immunohistochemistry

To detect virus antigen, sections were incubated with Rodent Block-M reagent (Biocare Medical, Pacheco, CA) for 20 minutes and incubated with a rabbit anti-SARS-CoV-2 nucleocapsid protein (NP) polyclonal antibody (abclonal, a18797), followed by an HRP-labeled goat anti-rabbit secondary antibody (Jackson ImmunoResearch Laboratories, 111-035-144). The sections were stained with the DAB Horseradish Peroxidase Chromogenic Kit (Servicebio, G1212) and visualized by a microscope.

### Statistical analyses

GraphPad Prism software was used for statistical analysis of data and graph generation. Data are expressed as mean and SEM. Normality was assessed using Shapiro-Wilk tests. Parametric tests were applied to normally distributed data, while non-parametric alternatives were used to non-normally distributed datasets. Unpaired two-tailed Student’s tests, Mann-Whitney *U* tests, or two-sided Wilcoxon tests were used for two-group comparisons. One-way ANOVA with Tukey’s multiple-comparison test and two-way ANOVA with Tukey’s multiple-comparison test were used for multi-group comparisons and two-factor comparisons. Welch’s correction was applied to *t*-tests when variances differed significantly. Pearson’s correlations were used for correlation analysis. The specific tests used for each experiment are detailed in the relative figure legends.

## Data Availability

The scRNA-seq data employed in this work are accessible at https://ngdc.cncb.ac.cn. They were deposited in the Genome Sequence Archive in the National Genomics Data Center, the China National Center for Bioinformation/Beijing Institute of Genomics, and the Chinese Academy of Sciences (CRA023259).

## SUPPLEMENTAL MATERIAL

Supplemental figures jvi.01231-25-s0001.docx Fig. S1 to S9.

ASM does not own the copyrights to Supplemental Material that may be linked to, or accessed through, an article. The authors have granted ASM a non-exclusive, world-wide license to publish the Supplemental Material files. Please contact the corresponding author directly for reuse.
