# Supplementary material for: Mucosal vaccination with long-form TSLP induces migratory cDC1-mediated adaptive immunity against SARS-CoV-2 infection
Source: J Virol. 2025 Aug 19;99(9):e01231-25. doi: 10.1128/jvi.01231-25 (PMC12456150; doi:10.1128/jvi.01231-25)
Supplement: Supplemental figures — Fig. S1 to S9. [file jvi.01231-25-s0001.docx]

**Supplementary information**

**Fig S1. lfTSLP and sfTSLP activate STAT5 in BMDCs depending on TSLPR.** (A) Bone marrow-derived DCs (BMDCs) from WT mice were treated with sfTSLP (100 ng/ml), lfTSLP (100 ng/ml), or mTSLP (100 ng/ml) for 5 or 15 minutes. Cell lysates were western blotted with antiphospho-STAT5 and anti-STAT5. (B) BMDCs from WT and *Tslpr*^–/–^ mice were treated with PBS, sfTSLP (100 ng/ml), lfTSLP (100 ng/ml), or mTSLP (100 ng/ml) for 15 minutes. Western blots of cell lysates were performed using antiphospho-STAT5 and anti-STAT5. β-actin was employed as a loading control.**
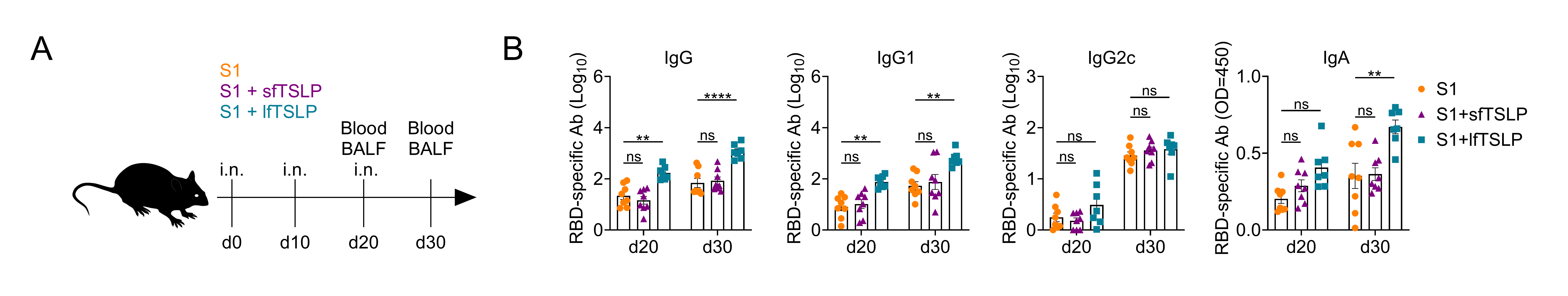
Fig S2. lfTSLP but not sfTSLP induce RBD-specific antibody production when S1 is applied by the intranasal route.** (A) WT mice were administered S1 in the presence or absence of sfTSLP or lfTSLP intranasally, collecting blood and BALF ten days after the first and second booster immunization. (B) The titers of RBD-specific IgG, IgG1, and IgG2c in serum and IgA in BALF were determined by ELISA. *n* = 7-8 animals per group. Experiments are shown as mean ± SEM. ns, no significant difference. ***P*<0.01, *****P*<0.0001, by two-way ANOVA with Tukey’s multiple-comparison test.

**
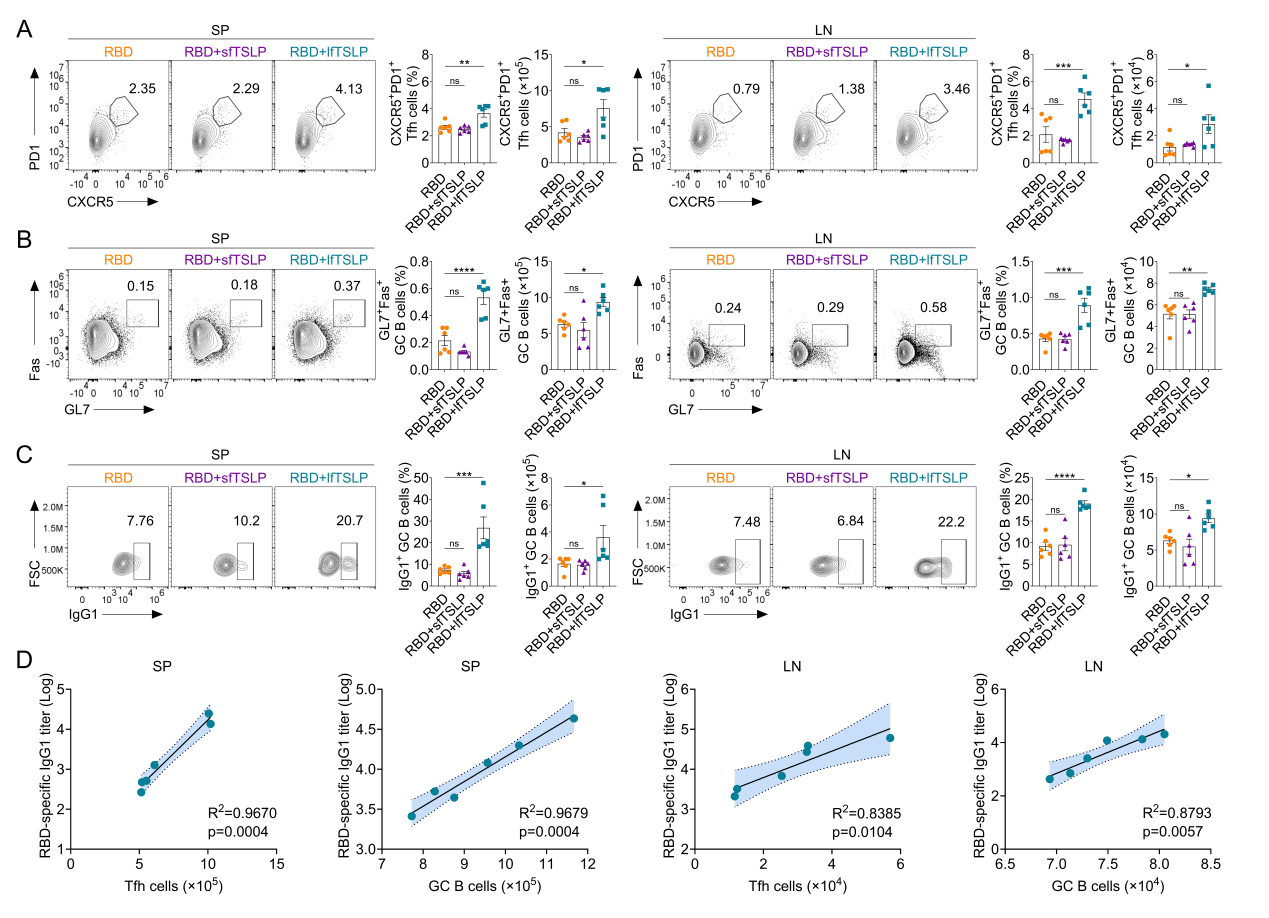
Fig S3. lfTSLP but not sfTSLP boosts Tfh cell and GC B cell responses when RBD is applied by the intranasal route.** WT mice were immunized by intranasal application of RBD alone or combined with sfTSLP or lfTSLP. *n* = 6 mice per group. Ten days after the third booster immunization, the SP and LN were analyzed by FACS for CXCR5^+^ PD-1^+^ Tfh cells among live CD4^+^ CD44^+^ cells (A), Fas^+^ GL7^+^ GC B cells among live CD19^+^ cells (B), and IgG1^+^ GC B cells among live CD19^+^ Fas^+^ GL7^+^ cells (C). (D) Correlation analysis between Tfh cell and GC B cell numbers of spleen (SP) and mediastinal lymph nodes (LN) and serum RBD-specific IgG1 titers in RBD and lfTSLP-immunized mice ten days after the third booster immunization. The Pearson correlation coefficient was used to determine the *r*-value for the correlation between the two groups. Results are representative of two independent experiments and are shown as mean ± SEM. **P*<0.05, ***P*<0.01, ****P*<0.001, *****P*<0.0001, by one-way ANOVA with Dunnett’s multiple-comparison test. ns, no significant difference.**
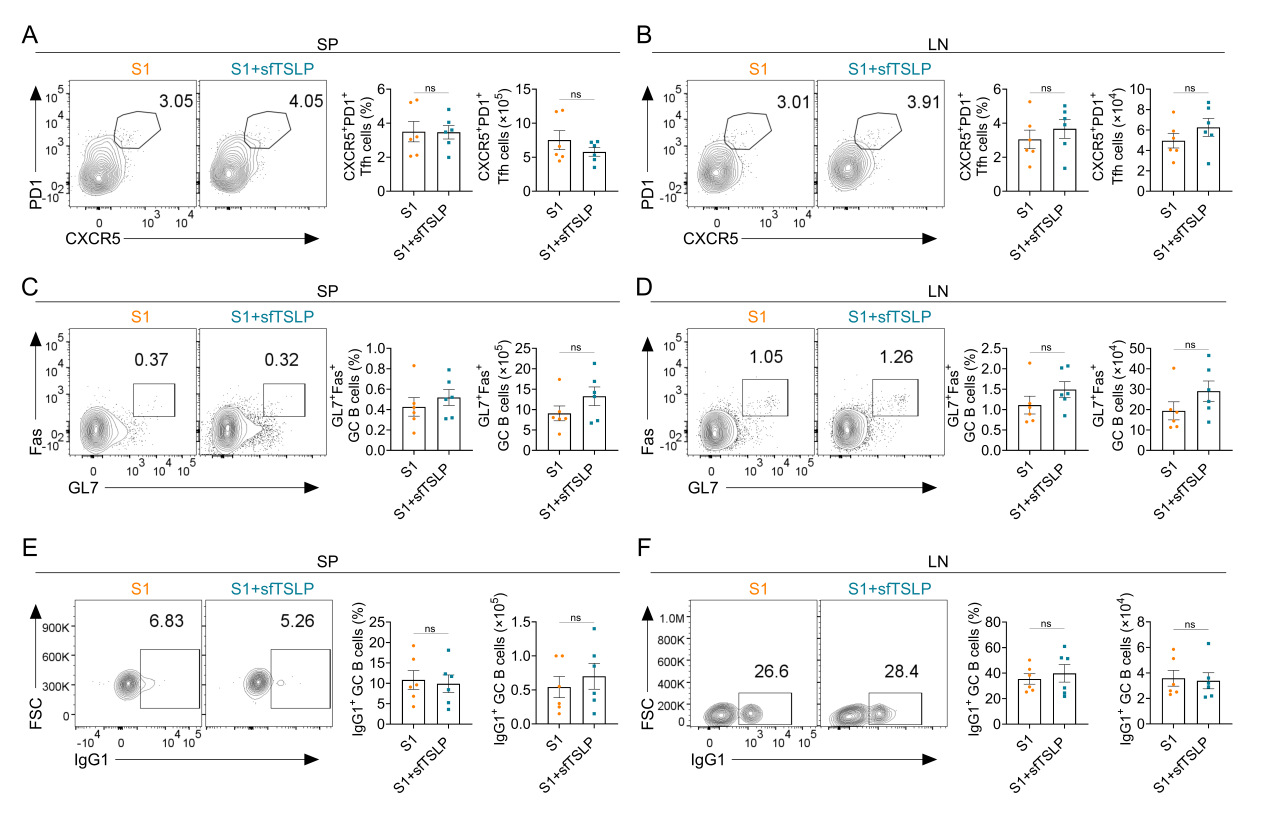
Fig S4. sfTSLP does not boost Tfh cells and GC B cell responses when S1 is applied by the intranasal route.** WT mice were immunized by intranasal application of S1 alone or combined with sfTSLP. *n* = 6 mice per group. Ten days after the second booster immunization, the spleen (SP) and mediastinal lymph nodes (LN) were analyzed by FACS for CXCR5^+^ PD-1^+^ Tfh cells among live CD4^+^ CD44^+^ cells (A-B), Fas^+^ GL7^+^ GC B cells among live CD19^+^ cells (C-D), and IgG1^+^ GC B cells among live CD19^+^ Fas^+^ GL7^+^ cells (E-F). Results are shown as mean ± SEM. ns, no significant difference, by unpaired two-tailed Student’s t-test (A-C, E-F) and Mann-Whitney *U*-tests (D).


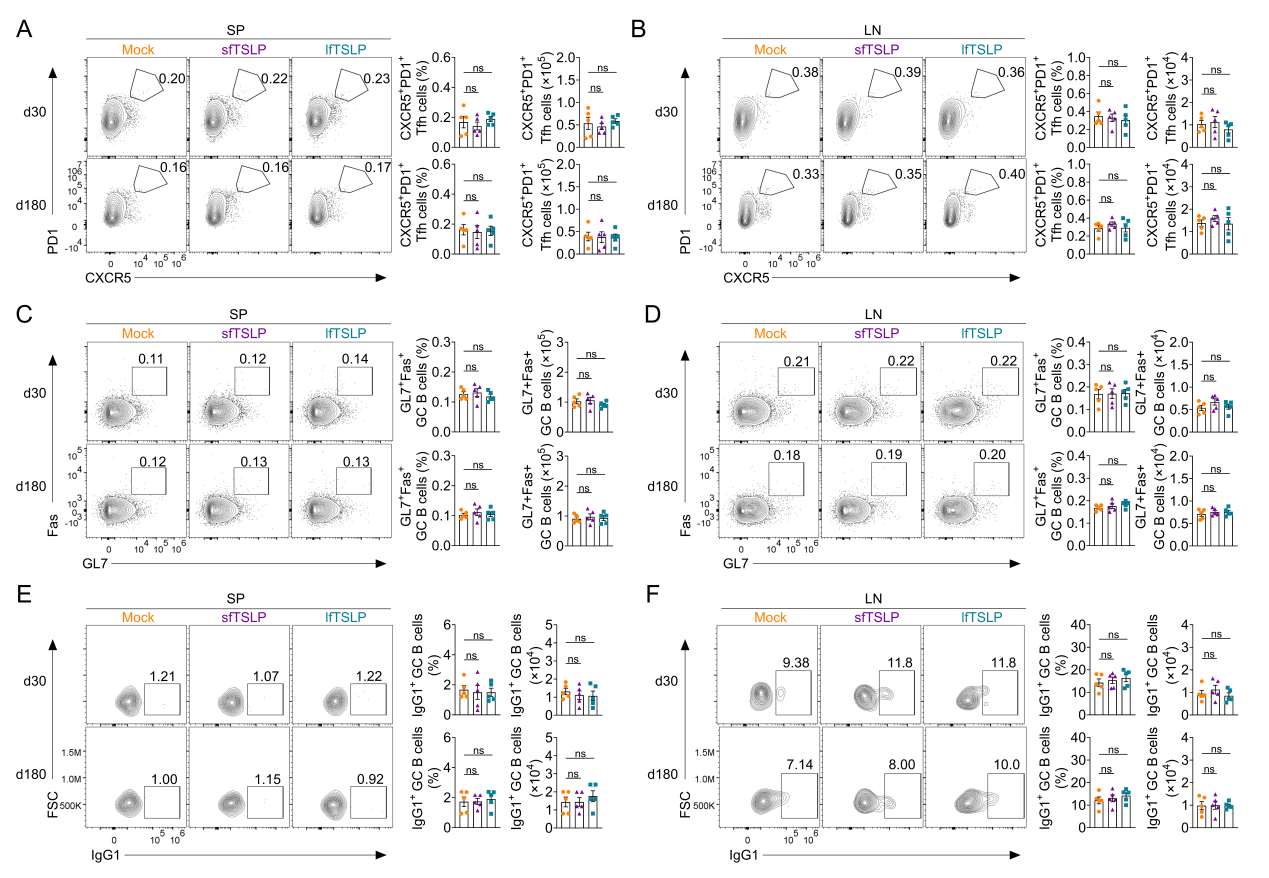


**Fig S5. Neither sfTSLP nor lfTSLP induces GC responses in the absence of antigen.** WT mice received intranasal sfTSLP or lfTSLP without antigen three times with ten-day intervals. Animals were sacrificed on days 30 and 180. *n* = 6 mice per group. The spleen (SP) and mediastinal lymph nodes (LN) were analyzed by FACS for CXCR5^+^ PD-1^+^ Tfh cells among live CD4^+^ CD44^+^ cells (A-B), Fas^+^ GL7^+^ GC B cells among live CD19^+^ cells (C-D), and IgG1^+^ GC B cells among live CD19^+^ Fas^+^ GL7^+^ cells (E-F). Results are shown as mean ± SEM. ns, no significant difference, by one-way ANOVA with Dunnett’s multiple-comparison test.

**
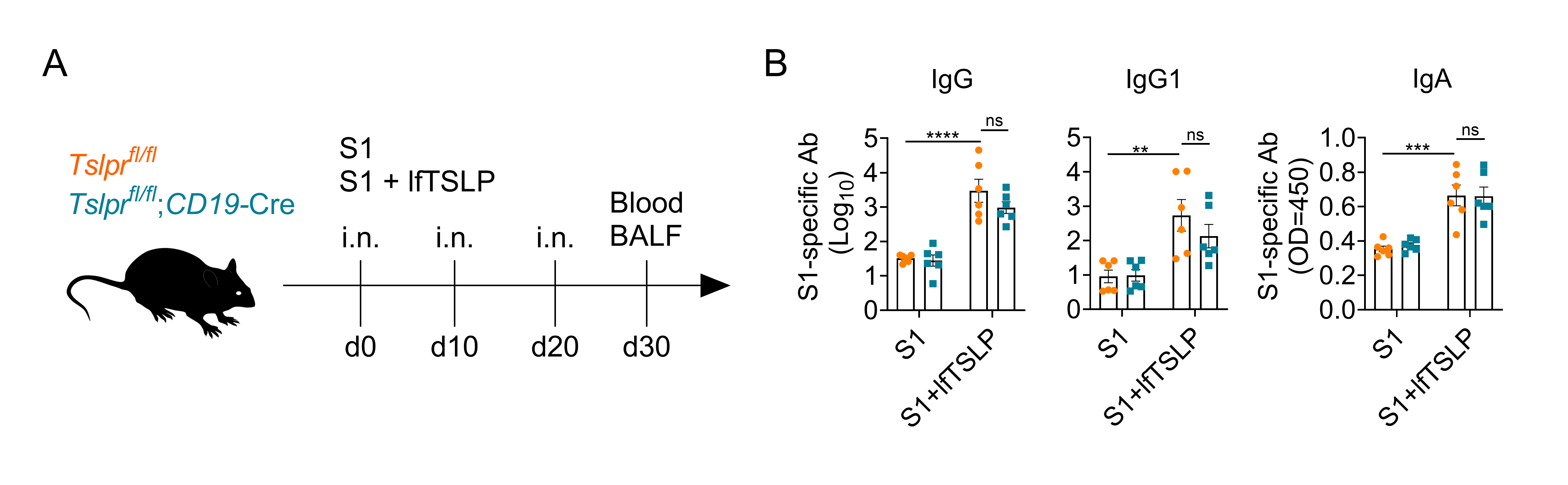
Fig S6. lfTSLP does not directly act on B cells to induce antibody production after intranasal immunization.** (A) *Tslpr^fl/fl^* (*n* = 6) and *Tslpr^fl/fl^*;*CD19*-Cre mice (*n* = 6) were intranasally immunized with S1 in the presence or absence of lfTSLP. Serum and BALF were collected 10 days after the second booster immunization. (B) S1-specific serum IgG and IgG1 titers and S1-specific BALF IgA titers were analyzed by ELISA. Results are shown as mean ± SEM. ***P*<0.01, ****P*<0.001, *****P*<0.0001, by two-way ANOVA with Tukey’s multiple-comparison test. ns, no significant difference.

**
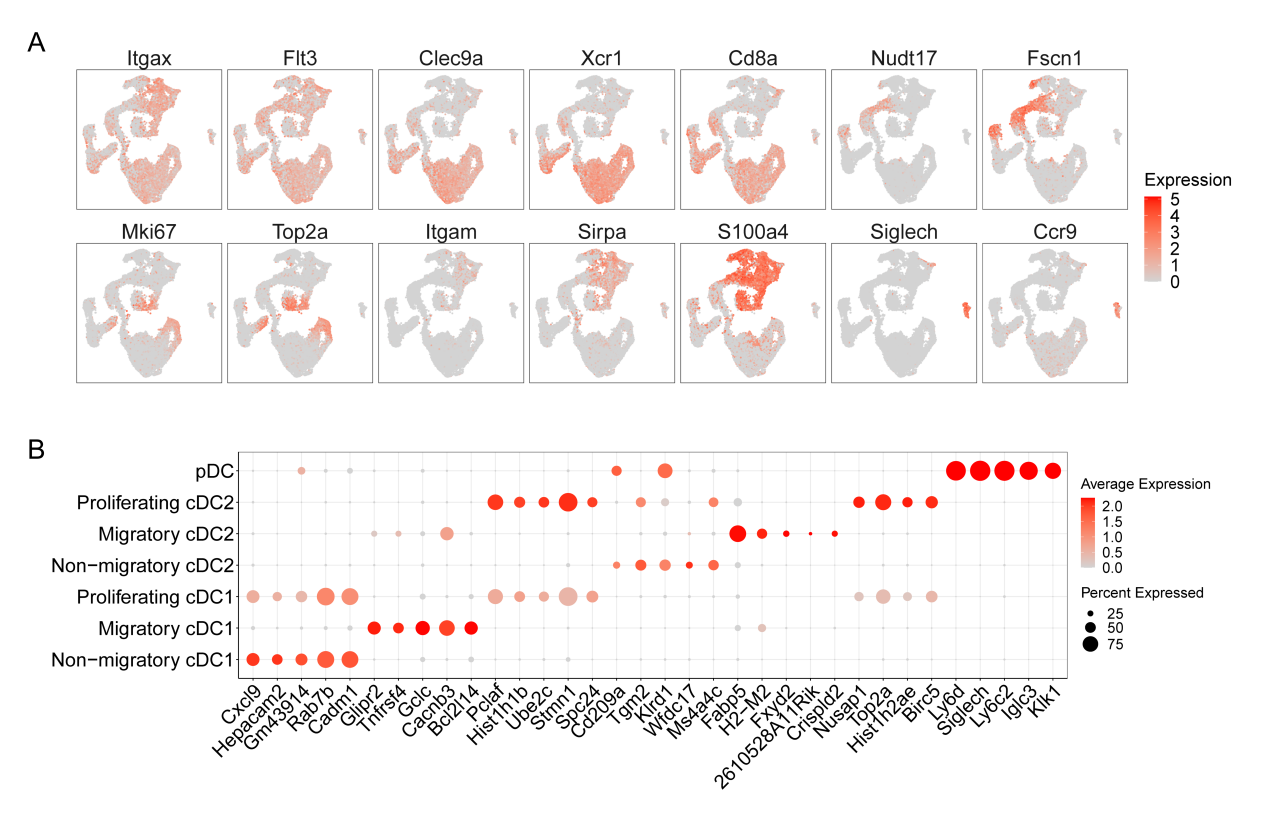
Fig S7. The expression of specific genes in different DC subtypes.** Single-cell RNA sequencing of different DC subtypes derived from pooled CD11c^+^ DCs of WT mice 5 days after intranasal S1 booster vaccination in the presence or absence of lfTSLP. (A) The expression of marker genes for different DC cell types. (B) The expression of the top 5 differentially expressed genes for each DC subtype. The dot color represents the average expression. The dot size represents the expression percentage.

**
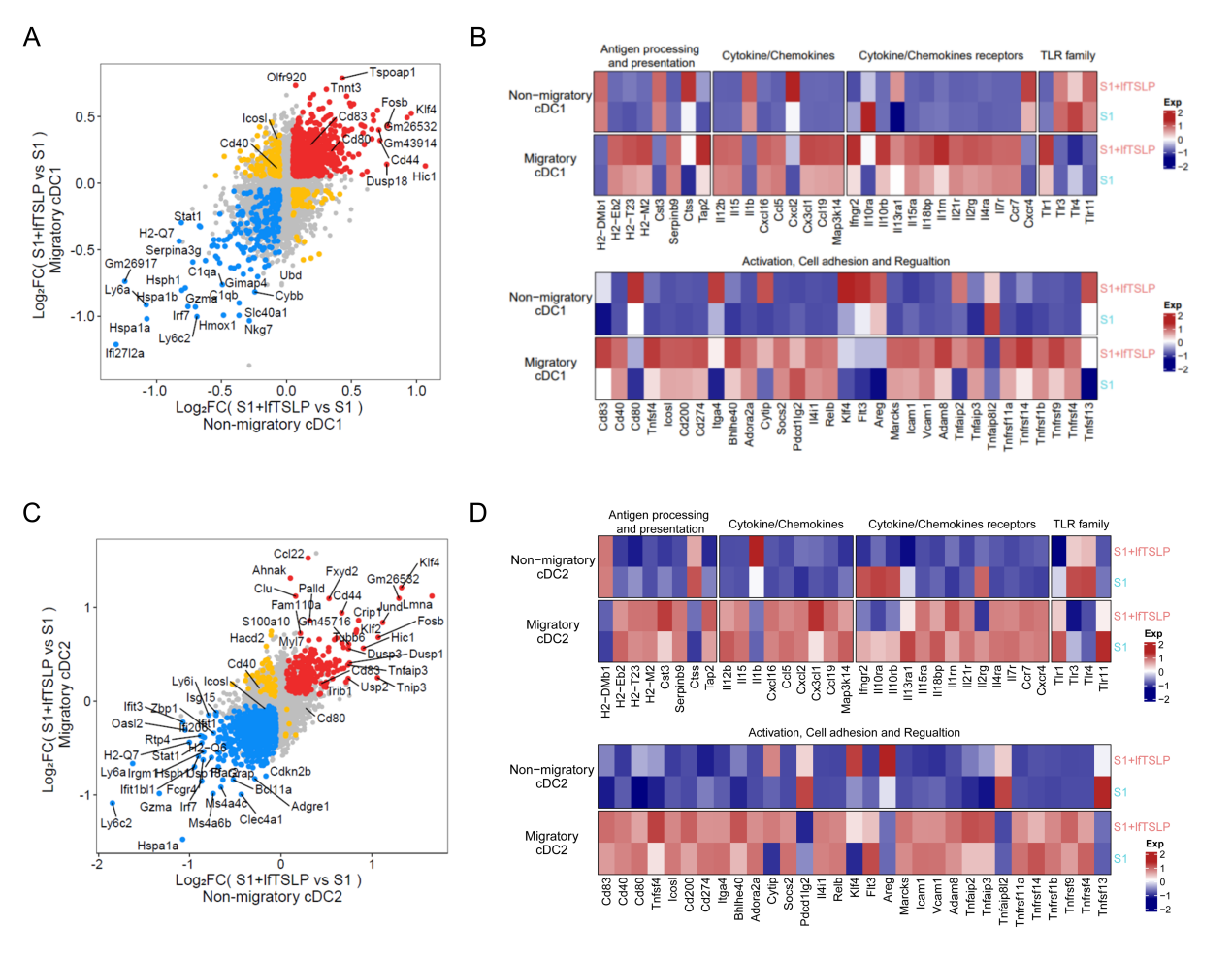
Fig S8. lfTSLP regulates distinct migratory and non-migratory cDC1 and cDC2 phenotypes.** Single-cell RNA sequencing analyses of migratory and non-migratory cDC1s and cDC2s isolated from pooled CD11c^+^ DCs of WT mice 5 days after booster immunization with S1 in the presence or absence of lfTSLP intranasally. (A) The scatter plot of DEGs between the S1 plus lfTSLP and the S1 group in migratory and non-migratory cDC1s (adjusted P value < 0.05). Red points represent up-regulated genes both in migratory and non-migratory cDC1s (Log_2_FC > 0.05); blue points represent down-regulated genes both in these two cell types (Log_2_FC < -0.05); yellow points represent up-regulated genes only in migratory or non-migratory cDC1s (Log_2_FC > 0.05); grey points represent genes with Log_2_FC < 0.05 or Log_2_FC > -0.05. FC: Fold change. (B) Heatmap showing the expression of specific DEGs between the S1 plus lfTSLP and the S1 group in migratory and non-migratory cDC1s. Color represents average expression. (C) The scatter plot of DEGs between the S1 plus lfTSLP and the S1 group in migratory and non-migratory cDC2s (adjusted P value < 0.05). Red points represent up-regulated genes both in migratory and non-migratory cDC2s (Log_2_FC > 0.05); blue points represent down-regulated genes both in these two cell types (Log_2_FC < -0.05); yellow points represent up-regulated genes only in migratory or non-migratory cDC2s (Log_2_FC > 0.05); grey points represent genes with Log_2_FC < 0.05 or Log_2_FC > -0.05. FC: Fold change. (D) Heatmap showing the expression of specific DEGs between the S1 plus lfTSLP and the S1 group in migratory and non-migratory cDC2s. Color represents average expression.

**
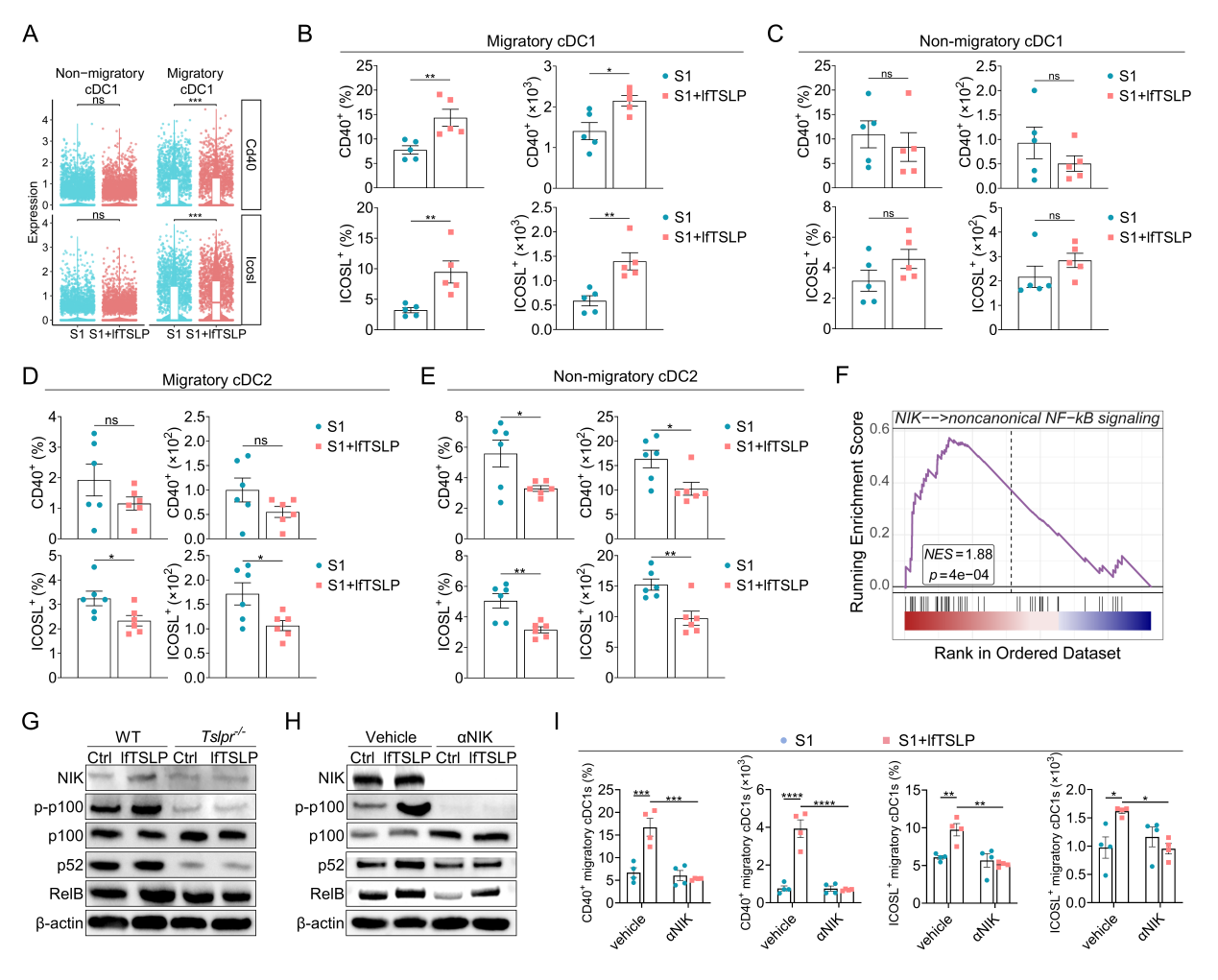
Fig S9. lfTSLP upregulates CD40 and ICSOL expression on migratory cDC1s via non-canonical NF-kB signaling.** (A-F) WT mice were intranasally immunized with S1 in the presence or absence of lfTSLP, and CD11c^+^ DCs were purified from LN on day 5 after the booster immunization for scRNA sequencing or FACS analysis. (A) The expression of CD40 and ICOSL between the S1 plus lfTSLP group and the S1 group in migratory and non-migratory cDC1s. *n* = 3 mice per group. The percentages and numbers of CD40 and ICOSL on migratory cDC1s (CD11c^+^ MHC-II^hi^ CD103^+^ CD11b^-^) (B), non-migratory cDC1s (CD11c^+^ MHC-II^low^ CD11b^-^ CD8^+^) (C), migratory cDC2s (CD11c^+^ MHC-II^hi^ CD103^-^ CD11b^+^) (D), and non-migratory cDC2s (CD11c^+^ MHC-II^low^ CD11b^+^ CD8^-^) (E) in LN were determined by FACS. *n* = 5 mice per group. (F) The NIK-->noncanonical NF-κB signaling pathway was significantly enriched in the S1 plus lfTSLP group in migratory cDC1s through GSEA analysis. (G-H) Migratory cDC1s from WT mice and *Tslpr^–/–^* mice were stimulated with or without lfTSLP in the presence of a vehicle or αNIK, and western blot was used to analyze the expression of molecules in the non-canonical NF-κB signaling pathway. (I) WT mice were immunized with S1 in the presence or absence of lfTSLP. Following immunization, mice were treated with a vehicle or αNIK, and the expression of CD40 and ICOSL on migratory cDC1s (CD11c^+^ MHC-II^hi^ CD103^+^ CD11b^-^) was detected by FACS. *n* = 4 per group. Results are shown as mean ± SEM. ns, no significant difference. **P*<0.05, ***P*<0.01, ****P*<0.001, *****P*<0.0001, by Wilcoxon test, two-sided (A), unpaired two-tailed Student’s t-test (B-E), and two-way ANOVA with Tukey’s multiple-comparison test (I).
